# Cellular taxonomy of Hic1^+^ mesenchymal progenitor derivatives in the limb: from embryo to adult

**DOI:** 10.1038/s41467-022-32695-1

**Published:** 2022-08-25

**Authors:** Martin Arostegui, R. Wilder Scott, Kerstin Böse, T. Michael Underhill

**Affiliations:** 1 Biomedical Research Centre, University of British Columbia, Vancouver, BC V6T 1Z3 Canada; 2 Department of Cellular and Physiological Sciences, University of British Columbia, Vancouver, BC V6T 1Z3 Canada; 3 School of Biomedical Engineering, 2222 Health Sciences Mall, University of British Columbia, Vancouver, BC V6T 1Z3 Canada

**Keywords:** Multipotent stem cells, Regeneration, Mesenchymal stem cells

## Abstract

Tissue development and regeneration rely on the cooperation of multiple mesenchymal progenitor (MP) subpopulations. We recently identified *Hic1* as a marker of quiescent MPs in multiple adult tissues. Here, we describe the embryonic origin of appendicular Hic1^+^ MPs and demonstrate that they arise in the hypaxial somite, and migrate into the developing limb at embryonic day 11.5, well after limb bud initiation. Time-resolved single-cell-omics analyses coupled with lineage tracing reveal that Hic1^+^ cells generate a unique MP hierarchy, that includes both recently identified adult universal fibroblast populations (*Dpt*^*+*^, *Pi16*^*+*^ and *Dpt*^*+*^ *Col15a1*^*+*^) and more specialised mesenchymal derivatives such as, peri and endoneurial cells, pericytes, bone marrow stromal cells, myotenocytes, tenocytes, fascia-resident fibroblasts, with limited contributions to chondrocytes and osteocytes within the skeletal elements. MPs endure within these compartments, continue to express *Hic1* and represent a critical reservoir to support post-natal growth and regeneration.

## Abstract (web summary)

Mesenchymal progenitors (MPs) are fundamental to tissue development and regeneration, however, their embryonic origin is unclear. Single cell-omics coupled with lineage tracing reveal a unique somite-derived MP hierarchy in limb morphogenesis.

## Introduction

Tissue-resident mesenchymal progenitors (MPs) can be found to varying extents in all tissues and play vital roles in tissue homoeostasis, renewal and regeneration^1,2^. Herein we use “MP” as an overarching term to capture the complete repertoire of progenitors with mesenchymal lineage potential. In adult appendicular skeletal muscle, heterogeneity within the MP population has been described, with a minimum of three distinct populations that exhibit restricted lineage potential^3,4^. After injury, a subset of these MPs, termed fibro/adipogenic progenitors (FAPs) and identified as *Pdgfra*^+^, *Ly6a*^+^, *Cd34*^+^, generate a transient population that orchestrates multiple facets of the regenerative programme. These include immune cell infiltration, vascularisation, trophic factor secretion, and both extracellular matrix (ECM) and basement membrane production and remodelling. Once an injury has resolved, “excess” FAPs are cleared via apoptosis and tissue integrity is restored^5,6^. Although *Pax7*^+^ muscle satellite stem cells are responsible for de novo myofiber synthesis, MPs and their derivatives are crucial components of the regenerative effort by secreting promyogenic cytokines and ECM that will orient and organise the developing myotubes^7–9^. Indeed, it has been shown that genetic ablation of FAPs results in skeletal muscle atrophy and impaired regenerative potential^10^. Based on homoeostatic transcriptomic profiles, FAPs can be subdivided into two major sub-groups, FAP1 and 2. The FAP1 cells express genes associated with the ECM, such as *Col4a1* and *a2*, various *Col6* transcripts, *Col15a1* and other ECM genes such as *Bgn*, *Mgp* and *Podn*. In contrast, FAP2 cells are enriched for transcripts involved in signalling and trophic factor production, such as *Dact2*, *Sfrp4*, *Wnt2*, *Sema3c*, *Pi16*, and *Tgfrbr2*. The FAP1 and 2 subsets correspond to the recently described two universal fibroblast profiles, *Dpt*^*+*^ *Col15a1*^*+*^ and *Dpt*^*+*^ *Pi16*^*+*^, described in mouse and human^4,11^.

The gene *Hypermethylated in Cancer 1* (*Hic1*) has been recently identified as a marker of adult tissue-resident MPs in skeletal muscle^4^, heart^12^, and skin^13^. HIC1 is a transcription factor and considered a putative tumour suppressor^14^ based in part on the observation that its overexpression was sufficient to induce growth arrest in various cell lines^15^. Furthermore, *HIC1* maps to the 17p13.3 chromosomal region that is often deleted or hypermethylated in human cancers^16^. Several HIC1 target genes have been identified, including cell cycle regulators, such as *CCND1*, *CDKN1A* and *CDKN1*C^17,18^. During murine embryonic development, *Hic1* has been shown to be expressed as early as E10.5 in the epaxial and hypaxial compartments of the somitic mesoderm and in various tissues thereafter^19^. *Hic1* null zygosity is associated with embryonic lethality at ~ embryonic day (E) 13.5 and embryos present with a spectrum of developmental abnormalities such as stunted growth, craniofacial deformities, and limb and digit dysmorphologies^20^.

In adult muscle, specialised Hic1^+^ MP subsets produce enduring progeny that regenerate fibrous connective tissues, which link muscle to bone, such as tenocytes and myotenocytes in addition to epimysium. *Hic1* also identifies laminin-encased *Rgs5*^*+*^, *Pdgfrb*^*+*^, *Kcnj8*^*+*^ and *Abcc9*^*+*^ pericytes. Indicative of their progenitor status, all of the described populations exhibit open chromatin regions (OCRs) across numerous cell cycle loci and enter the cell cycle following injury-induced activation^4^. MPs figure prominently in adult physiology and pathology, and Hic1^+^ MPs regulate multiple facets of the regenerative programme across multiple cell types^4,12,13^. However, our understanding of their embryonic origin and subsequent fates is unclear. The origin and fate of other tissue-resident stem and progenitor populations such as satellite cells within striated muscle have been well defined due to the specificity of the marker *Pax7*/PAX7. Fate mapping experiments performed in birds and mice have demonstrated that satellite cells, originate in the somitic dermomyotome and migrate to form the appendicular and axial skeletal musculature^21–23^, where they persist to form a reservoir of post-natal stem cells^24^. In contrast, due to the lack of effective markers of putative mesenchymal subsets in the early limb, it has been more challenging to resolve the origin, fate and cellular hierarchies within this compartment. In this study, we show that the early limb mesenchyme exhibits significant heterogeneity and that *Hic1* defines a distinct mesenchymal subset that contributes to limb fibroblasts and other specialised mesenchymal derivatives, some of which endure to support adult tissue regeneration.

## Results

### *Hic1* identifies a rare MP population within the limb bud

**Fig. 1:**
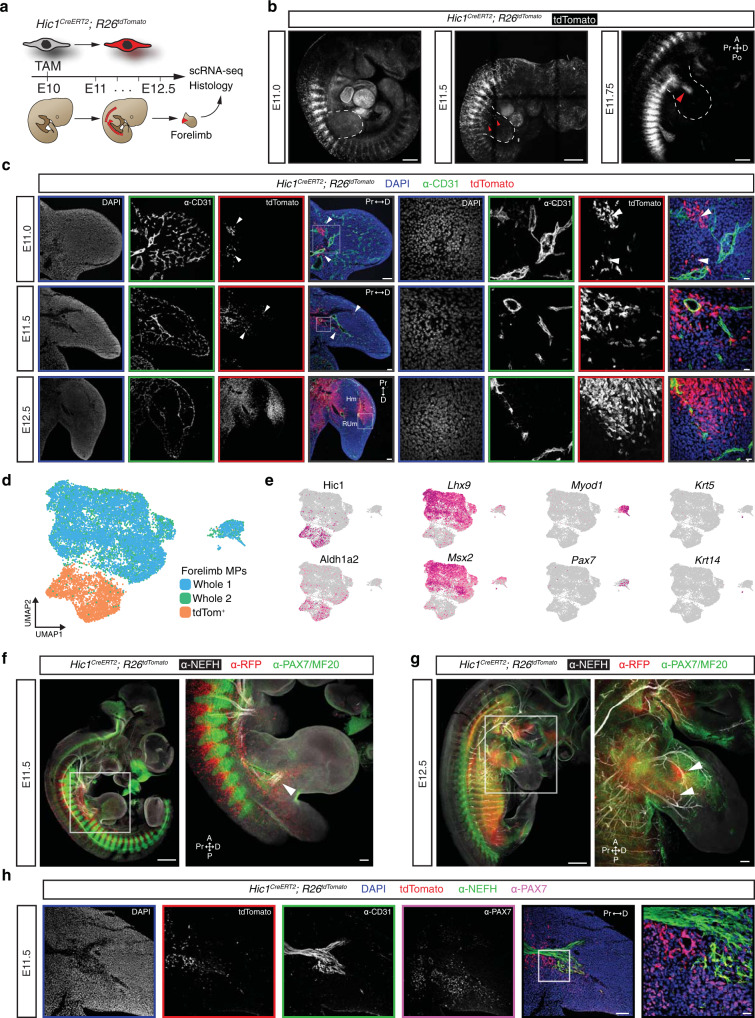
*Hic1* identifies a discrete population within the limb mesenchyme. **a** Schematic of the experimental strategy for *Hic1*^*CreERT2*^*; R26*^*tdTomato*^ genetic recombination. **b** Maximum intensity projections of whole-mount *Hic1*^*CreERT2*^; *R26*^*tdTomato*^ embryos. White-dotted-line outlines the forelimb; red arrowhead highlights tdTomato^+^ cells within the forelimb. *n* = 6 embryos from independent litters. Scale bar, 500 µm. **c** Representative images of *Hic1*^*CreERT2*^; *R26*^*tdTomato*^ forelimbs at sequential timepoints. *n* = 6 embryos from independent litters. Scale bars, 100 µm, 20 µm inset. White box represents inset region, white arrowheads define tdTomato^+^ cell clusters. **d** UMAP of aggregated scRNA-seq data representing whole forelimb dissociations (whole 1 and whole 2) and FACS-purified tdTomato^+^ MPs from E11.5 forelimb buds. **e** UMAPs of representative genes for each identified cell population: tdTomato^+^ MPs (*Hic1* and *Aldh1a2*), tdTomato^-^ MPs (*Lhx9* and *Msx2*), myogenic progenitors (*Myod1* and *Pax7*), and epithelium (*Krt5* and *Krt14*). **f** Maximum intensity projection of E11.5 *Hic1*^*CreERT2*^; *R26*^*tdTomato*^ embryo imaged and stained in whole-mount with antibodies against neurofilament heavy chain (NEFH), PAX7, and MF20. *n* = 3 embryos from independent litters. Scale bars, 500 µm and 100 µm. White box represents inset region highlighted in panel on right, white arrowhead indicates tdTomato^+^ cells within the limb. **g** Maximum intensity projection of E12.5 *Hic1*^*CreERT2*^; *R26*^*tdTomato*^ embryo stained in whole-mount with antibodies against NEFH, PAX7, and MF20. *n* = 3 embryos from independent litters. Scale bars, 500 µm and 100 µm. White box represents inset region highlighted in panel on right. Arrowheads indicate clusters of tdTomato^+^ cells. **h** Representative immunofluorescence micrographs of E11.5 *Hic1*^*CreERT2*^; *R26*^*tdTomato*^ forelimb sections stained with antibodies against NEFH and PAX7. *n* = 6 embryos from independent litters. Scale bars, 100 µm and 20 µm inset. White box represents inset region highlighted in bottom-right panel. A, anterior; D, distal; Po, posterior; Pr, proximal.

In the developing murine embryo, forelimb bud outgrowth initiates at approximately embryonic day (E)9^25^ and is composed of an ectodermal jacket encasing lateral plate mesoderm (LPM)-derived mesenchyme. Approximately 36 h post-initiation, *Hic1* transcripts become evident within the proximal limb mesenchyme^19^. Quantitative PCR analyses of E10.5 limb segments revealed low *Hic1* expression. In contrast, *Hic1* transcript was detected at E11.5 in a decreasing proximal to distal gradient (Supplementary Fig. 1a, b). To explore the nature and origin of Hic1^+^ cells in the limb, a lineage tracing strategy was employed involving a *Hic1*^*CreERT2*^ allele in conjunction with a Cre-dependent reporter, *R26*^tdTomato^ (Fig. 1a). A series of tamoxifen (TAM) pulse and chase experiments determined E10 to be the optimal time for the labelling of the emerging Hic1^+^ limb bud population (Supplementary Fig. 1c, d); these analyses also determined the TAM-labelling window to be 24–36 h. Embryos that were pulse-labelled with TAM at E10 showed no trace of tdTomato^+^ cells in the forelimb buds until ~E11.5 (Fig. 1b, c). Consistent with this, tdTomato^+^ cells were observed to be mostly restricted to the cultured proximal, but not distal, forelimb fragments and corresponding micromass cultures therefrom (Supplementary Fig. 1e, f).

To determine at which time the tdTomato^+^ cells populated the limb, E11 forelimb buds were analysed and the presence of two clusters at the body wall-limb bud interface was revealed (Fig. 1c, E11.0). By E11.75, whole-mount imaging identified a band of tdTomato^+^ cells within the hypaxial somite that extended into the proximal forelimb bud (Fig. 1b, E11.75 and Supplementary Movie 1). By E12.5, this band of cells extended to surround the chondrogenic core of the developing limb (Fig. 1c, E12.5 and Supplementary Fig. 1g, h). Flow cytometric (FCM) analysis at E11.5 showed that tdTomato^+^ cells had populated the central limb mesenchyme and constituted 0.45 ± 0.16% of total forelimb mesenchymal cells (Supplementary Fig. 1i). The frequency of this population increased markedly to comprise 5.79 ± 1.1%, 12.1 ± 1.6%, and 26.6 ± 0.5% of the limb mesenchymal cell compartment by E12.5, E13.5 and E14.5, respectively (Supplementary Fig. 1i). To determine if tdTomato^+^ cells represented a unique subset of mesenchymal cells, single-cell RNA-sequencing (scRNA-seq) was performed on E11.5 forelimb buds. TdTomato^+^ cells clustered separately from other whole-limb mesenchymal cells (Fig. 1d). Genes such as *Hic1* and *Aldh1a2* distinguished the tdTomato^+^ cluster from tdTomato^-^ limb bud mesenchymal cells (*Lhx9*^*+*^*, Msx2*^*+*^), myogenic precursors (*Myod1*^*+*^*, Pax7*^*+*^) and epithelium (*Krt5*^*+*^*, Krt14*^*+*^) (Fig. 1e). Co-expression of the retinoic acid (RA) synthesis gene, *Aldh1a2* with *Hic1* is consistent with the direct regulation of *Hic1* transcription by the nuclear receptors for RA, RARs^26^. The differential expression signature of tdTomato^+^ cells further reinforced the distinct nature of this population. At E11.5, tdTomato^+^ cells were observed in close association with migrating myogenic progenitors and projecting nerve axons (Fig. 1f, h). One day later, at E12.5, tdTomato^+^ cells were interspersed amongst the developing dorso-ventral muscle masses and their accompanying nerves (Fig. 1g). Indeed, immunofluorescence (IF) analysis at E13.5 revealed abundant tdTomato^+^ cells within the dorsal and ventral muscle masses underlying the epithelium and circumscribing the developing chondrogenic anlagen (Supplementary Fig. 1k). However, the tdTomato^+^ cells did not contribute to the primary cartilaginous elements, which are derived from the LPM (Figs. 1c, E12.5 and Supplementary Fig. 1k). Furthermore, expansion of the tdTomato^+^ compartment was associated with a proliferative phase in which up to 45.2 ± 2.1% (E12.5) of the tdTomato^+^ cells had incorporated EdU (Supplementary Fig. 1j).

The pattern of migration of tdTomato^+^ cells was consistent with other migratory populations, especially myogenic progenitors, which derive from the somite. To investigate the origin of tdTomato^+^ cells, the distribution of tdTomato^+^ cells was compared to markers reflective of the distinct somitic compartments including, the myotome (PAX3, MYOD1) and sclerotome (PAX1), as well as neural crest-derived cells (SOX10). As mentioned previously, tdTomato^+^ MPs do not co-localise with PAX7^+^ cells; however, myoblasts that are actively migrating during early forelimb morphogenesis strongly express *Pax3*^27^. A subset of limb pericytes and vascular smooth muscle cells have been found to derive from the somitic sclerotome^28^ and therefore α-PAX1 was used to identify the sclerotome at the time of tdTomato-cell migration. Lastly, α-SOX10 was chosen to label neural-crest-derived migratory cells^29^. TdTomato^+^ cells showed no overlap with migratory PAX3^+^ myotome-derived progenitors (Supplementary Fig. 2a), MYOD1^+^ myogenic progenitors (Supplementary Fig. 2b), nor with SOX10^+^ neural crest-derived cells (Supplementary Fig. 2c). Interestingly, a small number of PAX1/tdTomato double-positive cells were observed (Supplementary Fig. 2d). Furthermore, the sclerotome contained a population of *Scx*^*+*^ tenogenic progenitors (Scx-GFP), which also overlapped with tdTomato^+^ cells at the forelimb level (Supplementary Fig. 2d). In summary, these results indicate that tdTomato^+^ MPs are migratory cells that do not derive from the dermomyotome nor neural crest, but originate within the sclerotome and syndetome compartments of the somite.

**Fig. 2:**
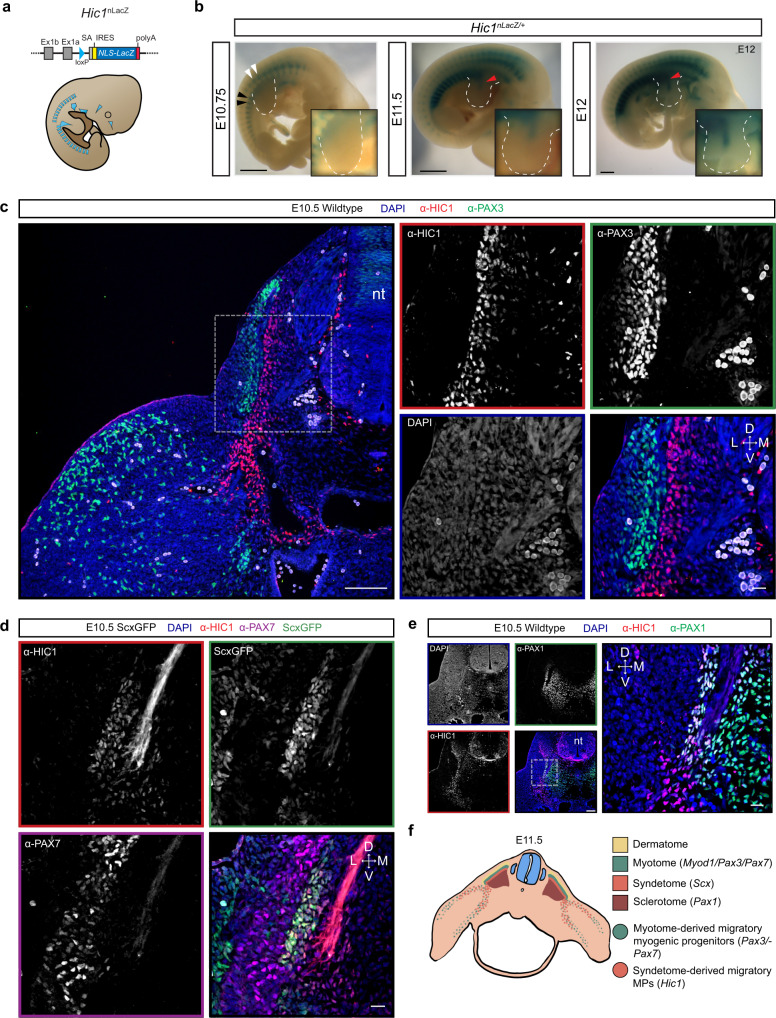
*Hic1*/HIC1 is expressed in the syndetome and sclerotome of developing somites. **a** Schematic of *Hic1*^*nLacZ*^ allele. **b** Representative whole-mount X-gal staining of *Hic1*^*+/nLacZ*^ embryos at the indicated time points. Forelimb buds outlined in white. Arrowheads: Black- hypaxial sclerotomes, white- epaxial sclerotomes, red- *Hic1* expression within the central region of forelimb bud. Scale bars, 1 mm. **c** Immunofluorescence staining with α-HIC1 and α-PAX3 on E10.5 embryos at the forelimb level. Dotted box highlights area inset in adjoining panels. *n* = 3 embryos from independent litters. Scale bars, 100 µm, 20 µm inset. **d** Immunofluorescence staining with α-PAX7 on E10.5 Scx-GFP embryos. *n* = 3 embryos from independent litters. Scale bar, 20 µm. **e** Immunofluorescence staining with α-HIC1 and α-PAX1 on E10.5 embryos. Dotted box highlights area inset in adjoining panel. *n* = 3 embryos from independent litters. Scale bars, 100 µm, 20 µm inset. D, dorsal; L, lateral; M, medial; nt, neural tube; V, ventral. **f** Schematic representation of Hic1^+^ MP migration from the somite.

To further elucidate the embryonic origin of Hic1^+^ MPs, a *Hic1*^*nLacZ*^ reporter mouse model, in combination with α-HIC1 antibody staining, were used to identify Hic1^+^ MPs prior to E11.5 (Supplementary Fig. 2e). The *Hic1*^*nLacZ*^ reporter mice enabled visualisation of the spatial distribution of Hic1^+^ cells in whole-mount embryos (Fig. 2a) where by E10.75, X-gal staining was evident within regions of the epaxial and hypaxial somitic mesenchyme (Fig. 2b). At E11.5, LacZ^+^ cells were observed within the proximal limb mesenchyme (Fig. 2b). At this time, *Hic1* exhibits limited and regionalised expression in the limb and is restricted to the proximal mesenchyme. Subsequently, X-gal^+^ cells were observed within the central proximal region by ~E12 (Fig. 2b). At later stages, Hic1^+^ cells were visualised throughout the limb mesenchyme and circumscribed various chondrogenic anlagen (Supplementary Fig. 2f). Consistent with the interstitial distribution of the Hic1^+^ MP population in adult muscle^4^, Hic1^+^ cells could also be found within the emerging ventral and dorsal muscle masses (Supplementary Fig. 2f). Similarly, direct detection of the HIC1 protein with α-HIC1 revealed that at E10.5, Hic1^+^ MPs were located within the somitic mesenchyme. Specifically, Hic1^+^ MPs were medial to the PAX3^+^ myotome and juxtaposed with migrating PAX3^+^ myogenic progenitors at the hypaxial end of the somite, in close proximity to the proximal edge of the forelimb bud (Fig. 2c). As expected, HIC1^+^ cells largely overlapped with the ScxGFP^+^ syndetome (Fig. 2d) and exhibited partial overlap with the lateral portion of the PAX1^+^ sclerotome (Fig. 2e). Collectively, these results indicate that Hic1^+^ MPs are distinct from PAX3^+^ myogenic progenitors, originate within the sclerotomal/syndetomal compartments of the somite (Fig. 2f) and support the concept that Hic1^+^ MPs represent a unique migratory MP subset within the developing limb.

### scRNA-seq and histological analyses reveals a unique MP hierarchy

**Fig. 3:**
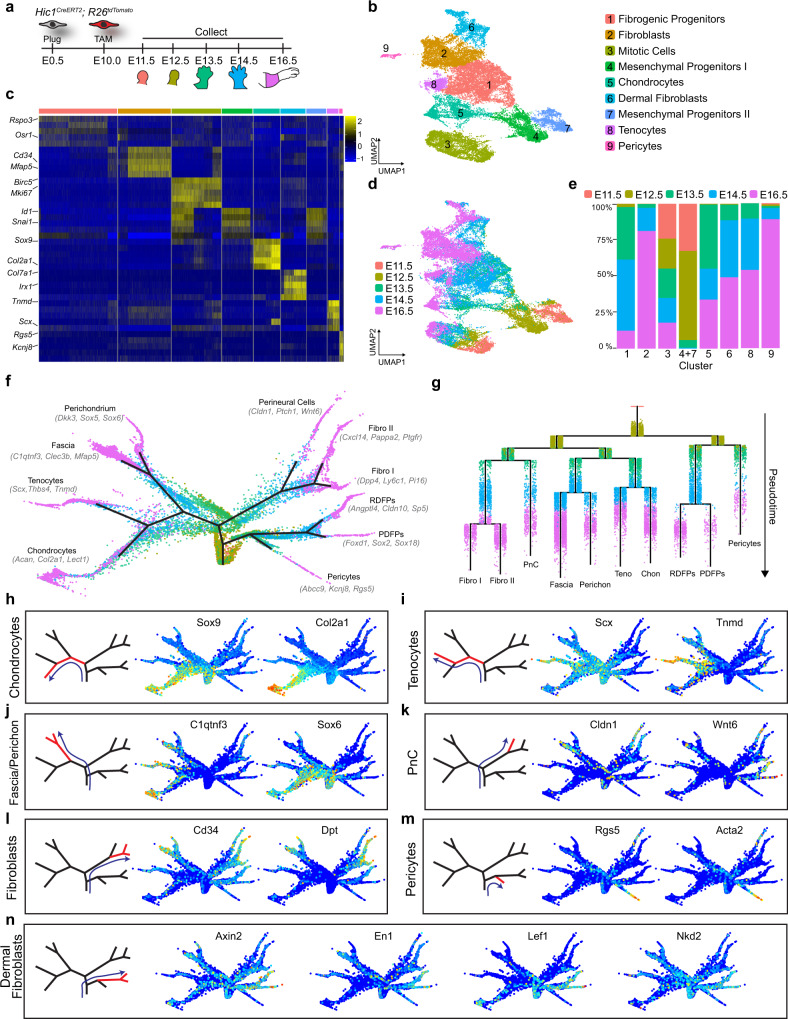
ScRNA-seq analyses define a mesenchymal cell progenitor hierarchy. **a** Schematic of *Hic1*^*CreERT2*^; *R26*^*tdTomato*^ sample collection for scRNA-seq. **b** UMAP of aggregated scRNA-seq data from sorted forelimb tdTomato^+^ cells coloured/numbered according to cluster identity determined by Louvain clustering. **c** Heatmap of scRNA-seq data showing enriched genes within each cluster. **d** UMAP of aggregated scRNA-seq data from sorted forelimb tdTomato^+^ cells coloured according to embryonic timepoint. **e** Stacked bar graph illustrating the composition of each cluster with respect to embryonic timepoints. **f** Force-directed layout of Hic1-lineage-labelled MP differentiation trajectory, coloured according to developmental timepoint and optimised for 2D visualisation. **g** URD-inferred developmental trajectories of Hic1-lineage-labelled forelimb MPs, coloured by developmental timepoint. Chon, chondrocytes; fibro, fibroblasts; PDFPs, papillary dermal fibroblast progenitors; perichon, perichondrium; PnC, perineural cells; RDFPs, reticular dermal fibroblast progenitors; teno, tenocytes. **h**–**n** Representative gene expression heatmaps projected on layout from (**f**), showing transcript abundance of specified mesenchymal cell fate markers. Blue lines in each plot indicate trajectory and red branch highlights tip/cell fate. Source data are provided as a Source Data file.

To characterise the molecular nature and developmental fates of tdTomato^+^ cells, scRNA-seq was performed on FACS-purified limb populations at 5 developmental timepoints ranging from E11.5 to E16.5 (Fig. 3a and Supplementary Data 1). In total, 32,225 cells were profiled. Louvain-Jaccard clustering identified 9 distinct cell populations within the aggregated dataset and select cell-type markers were used to annotate the clusters (Fig. 3b, c). Chronologically, the earliest populations (MPI, MPII) were composed mainly of cells from E11.5 (32%) and E12.5 (62%) (Fig. 3d, e) and expressed progenitor cell markers such as *Id* genes^30^ (Fig. 3c), as well as genes involved in cell migration and locomotion. Mitotic cells (MCs) formed a distinct cluster enriched for proliferation-associated genes such as *Birc5*, *Mki67*, and *Ccnb1* (Fig. 3c). The majority of cells from E14.5 and E16.5 expressed cell lineage-defining markers indicative of a more differentiated phenotype. They formed six different groups based on gene expression: fibroblasts (*Fap*, *Fbn1*), chondrocytes (*Lect1*, *Col2a1*), dermal fibroblasts (*Col7a1*, *Rspo1*), tenocytes (*Tnmd*, *Htra3*), and pericytes (*Rgs5*, *Kcnj8*) (Fig. 2b, c). An intermediate cluster between MPs and fibroblasts was termed “fibrogenic progenitors” and expressed markers such as *Osr1*, *Rspo3*, and *Hmcn1* (Fig. 3b, c). As the E16.5 forelimb is relatively large, scRNA-seq was carried out on two separate domains in an attempt to capture the totality of Hic1^+^ MP lineage contribution (Supplementary Fig. 3a). In total 7758 distal and 9275 proximal tdTomato^+^ cells were aggregated and analysed (Supplementary Fig. 3b). For the most part, cells within these two compartments exhibited similar lineage fates. However, given their distribution along the proximal-distal axis, genes associated with positional identity (i.e., Hox genes) exhibited differential expression between the proximal and distal sampled tdTomato^+^ cells (Supplementary Fig. 3c). Louvain-Jaccard clustering revealed 7 cell types, which were annotated based on marker gene expression (Supplementary Fig. 3d). Fibroblasts (*Cd34*^+^, *Dpt*^+^), dermal fibroblasts (*Irx1*^+^, *Col7a1*^+^), chondrocytes (*Sox9*^+^, *Col2a1*^+^), tenocytes (*Scx*^+^, *Tnmd*^+^), and pericytes (*Rgs5*^+^, *Acta2*^+^) were identified as distinct cell types (Supplementary Fig. 3e, f). Overall, cell types were represented to similar extents within the proximal and distal limb compartments (Supplementary Fig. 3g).

To further refine the tdTomato^+^ cell lineage tree, the URD analytical tool^31^ was used to generate differentiation trajectories for the aggregated (E11.5–E16.5) dataset. The tips, or terminal populations, were manually curated based on marker analysis (Supplementary Fig. 4a, b; Supplementary Data 2). The reconstructed trajectory tree recapitulated the identified clusters and effectively ordered each of the populations from the intermediate timepoints (E12.5, E13.5, E14.5) in chronological order without a priori annotation (Fig. 3f, g). The tree segments closest to the root represent earlier stages on the pseudotime trajectory and were enriched for MP cell markers such as *Hmga2*, *Id1*, *Osr1*, *Snai1*, *Sox11, and Tcf7l2* (Supplementary Fig. 4d). The developmental trajectories that emanated from the E11.5 tdTomato^+^ population were resolved into 8 major distinct fates: chondrocytes (*Sox9*, *Col2a1;* Fig. 3h)^32^, tenocytes (*Scx*, *Tnmd;* Fig. 3i)^33^, fascia (*C1qtnf3, Mfap5, Clec3b;* Fig. 3j and Supplementary Fig. 5a, b)^34^, periskeletal cells (perichondrium, *Sox5, Sox6, Dkk3*; Fig. 3j and Supplementary Fig. 5c)^35^, perineurial cells (*Cldn1, Wnt6*; Fig. 3k*)*^36^, fibroblasts (*Cd34*, *Dpt*; Fig. 3l and Supplementary Fig. 5d, e)^35,37^, pericytes (*Rgs5*, *Acta2*; Fig. 3m)^38^ and dermal fibroblasts (*Axin2, En1, Lef1, Nkd2*; Fig. 3n and Supplementary Fig. 5f, g)^39,40^.

**Fig. 4:**
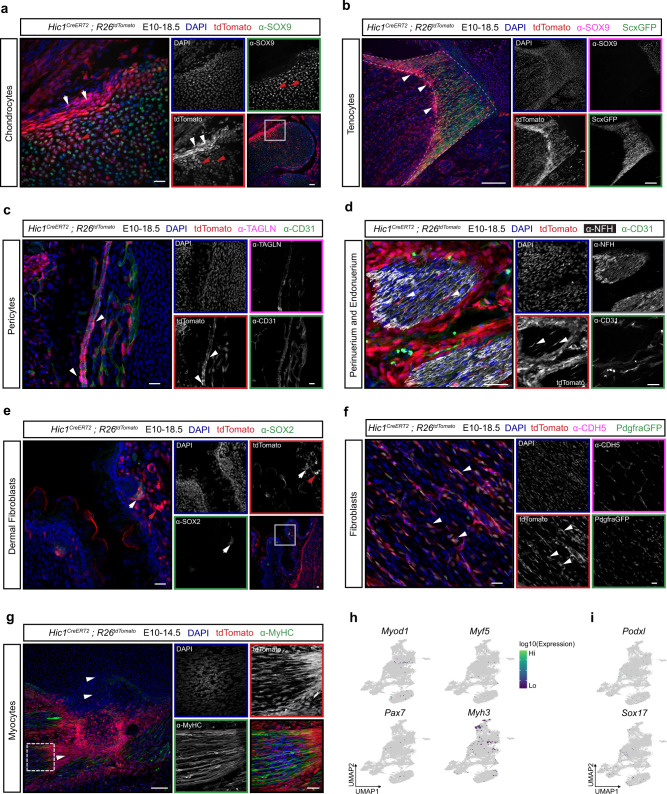
Lineage tracing analyses confirm computationally inferred lineage potential. **a**–**f** Representative immunofluorescence images of E18.5 *Hic1*^*CreERT2*^; *R26*^*tdTomato*^ forelimbs of tdTomato^+^ MP-derived cells demonstrating contribution to the following lineages: chondrocytes and perichondrium. White arrowheads indicate perichondrium, red arrowheads highlight tdTomato^+^ chondrocytes; **b** tenocytes; **c** pericytes; **d** perineurium and endoneurium cells. White arrowheads indicate endoneurial cells; PDFPs and RDFPs highlighted by white and red arrowheads respectively (**e**); and fibroblasts (**f**). White boxes in **a**, **e**, **g** highlight regions of interest that are magnified in adjoining panels. Scale bars, 20 µm. **g** Representative immunofluorescence image of an E14.5 *Hic1*^*CreERT2*^; *R26*^*tdTomato*^ embryo (TAM at E10) stained with α-MyHC. Scale bar, 100 µm. *n* = 6 embryos from independent litters for panels **a**–**g**. **h** UMAP plots of aggregated scRNA-seq data from *Hic1*^*CreERT2*^; *R26*^*tdTomato*^-sorted tdTom^+^ cells showing no detectable expression of myogenic regulatory factors (*Myod1*, *Myf5*, and *Pax7*) and embryonic myosin (*Myh3*). **i** UMAP plots of aggregated scRNA-seq data from *Hic1*^*CreERT2*^; *R26*^*tdTomato*^-sorted tdTomato^+^ cells showing no detectable expression of endothelial cell markers (*Podxl* and *Sox17*).

To experimentally interrogate the computationally inferred lineage trajectories, the fate of tdTomato^+^ cells was examined in fetal forelimbs. By E18.5, tdTomato^+^ clusters of chondrocytes were observed concentrated in specific areas of the developing bones, such as the distal humerus (Fig. 4a). TdTomato^+^ ScxGFP^+^ tenocytes were found mostly in short limb tendons, for example at the connection point between the biceps brachii and the radius (Fig. 4b). Numerous stromal compartments containing varying contributions of tdTomato^+^ cells in alignment with the in silico-derived lineages were also observed. TdTomato^+^ TAGLN^+^ cells, indicative of pericytes associated with capillaries^38^, were identified throughout the E18.5 forelimb (Fig. 4c). Similarly, tdTomato^+^ cells were identified circumscribing and within nerve axon bundles of the appendicular peripheral nervous system (Fig. 4d). Numerous tdTomato^+^ cells populated connective tissues of the limb skin including SOX2^+^ dermal papilla fibroblasts and SOX2^-^ reticular fibroblasts^41,42^ (Fig. 4e). In skeletal muscle, tdTomato^+^ Pdgfra^+^ fibroblasts were abundant within the fascia, skeletal muscle interstitium and associated connective tissues^9^ (Fig. 4f). However, tdTomato^+^ cells did not contribute to the myogenic (Fig. 4g, h) nor endothelial lineages (Fig. 4i). Collectively, these findings reinforce that early Hic1^+^ MPs represent a hitherto undescribed mesenchymal cell population with multi-lineage potential.

### Multi-lineage potential of Hic1^+^ MPs is embedded in the chromatin landscape

**Fig. 5:**
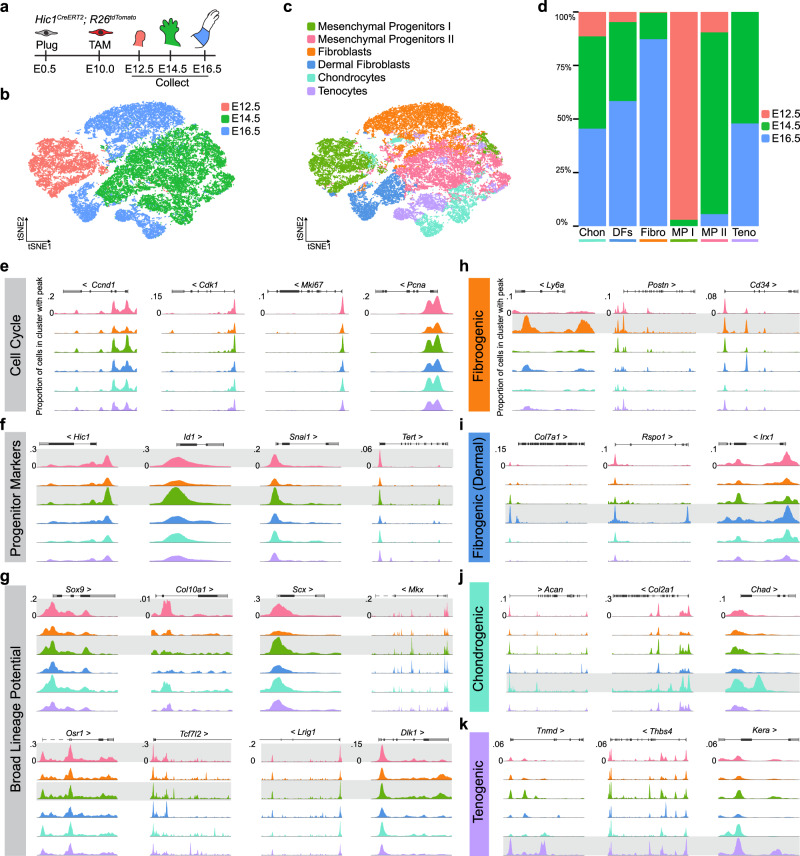
Broad mesenchymal lineage potential is embedded in the epigenome of early tdTomato^+^ MPs. **a** Schematic of *Hic1*^*CreERT2*^; *R26*^*tdTomato*^ sample collection for scATAC-seq. **b** t-SNE plot of aggregated scATAC-seq data from sorted tdTomato^+^ MPs coloured according to embryonic timepoint. **c** t-SNE plot of aggregated scATAC-seq data from sorted tdTomato^+^ MPs coloured according to cluster identity. **d** Stacked bar graph illustrating the composition of each cluster with respect to embryo timepoints. **e**–**k** Genome browser tracks from scATAC-seq analyses displaying the promoter sum signal near the indicated gene loci. Colours for each track correspond to cluster identities defined in panel **c**. Grey boxes highlight clusters exhibiting promoter sum signal relevant to the indicated cell function or fate. Chon, chondrocytes; DF, dermal fibroblasts; Fibro, fibroblasts, MP I/II, mesenchymal progenitors I/II, Teno, tenocytes. Source data are provided as a Source Data file.

To further interrogate the lineage potential underlying the proposed cell hierarchy, epigenetic profiling using scATAC-seq was performed on a total of 41,066 single nuclei from sorted tdTomato^+^ forelimb cells captured at E12.5, E14.5, and E16.5 (Fig. 5a, b). *K*-means clustering (*k* = 7) projected onto a *t*-stochastic neighbour-embedding (*t*SNE) dimensionality reduction plot identified 6 main cell populations (Fig. 5c). This analysis yielded two MP clusters: MPs I and II. The majority of cells comprising MPs I were derived from the E12.5 limb sample (97%); similarly, cells from MPs II were mostly derived from E14.5 limbs (85%) (Fig. 5d). MPs were found to have OCRs at gene loci associated with the cell cycle and progenitor status, such as *Hic1*, *Id1* and *Snai1* (Fig. 5e, f)^4,30,43^. OCRs within lineage-defining genes corresponding to the aforementioned 8 major cell fates were all evident within these populations (Fig. 5g). For example, OCRs were detected at gene ranges encompassing the *Sox9* (chondrogenic), *Scx* (tenogenic), *Osr1* (fibro/adipogenic)^44^, and *Lrig1* (dermal fibrogenic) loci (Fig. 5g) indicative of broad mesenchymal lineage potential. With time, loci associated with alternative cell fate trajectories became differentially accessible as lineage-restricted populations emerged (Fig. 5h–k).

The largest population was identified as a collection of fibrogenic-cell types with OCRs identified in loci including *Ly6a*, *Postn*, and *Cd34* (Fig. 5h). A dermal fibrogenic cluster consisted of cells containing OCRs within the *Col7a1*, *Rspo1*, and *Irx1* loci (Fig. 5i)^40,45,46^. The chondrogenic and tenogenic progenitor clusters were unambiguously classified based on OCRs within *Acan*, *Col2a1*, and *Chad*, or *Tnmd*, *Thbs4*, and *Kera* loci, respectively (Fig. 5j, k). Distinct pericyte and perineural clusters were not detected, although respective underlying OCRs (*Kcnj8, Rgs5, Cldn1, Wnt6*) were identified and these populations were found to be interspersed throughout the clusters. This likely reflects the fact that these rare populations are less efficiently resolved at these time points. As would be expected from a multipotent cell population upstream of a lineage trajectory, limited transcription from OCRs associated with the committed mesenchymal lineages was observed in MPs at E12.5 (Supplementary Fig. 6a, b). This potential was only realised in more committed progenitors and differentiated cells at E16.5 with the detection of the corresponding transcripts (Supplementary Fig. 6c), indicating that Hic1^+^ MP multipotency is embedded within the epigenome.

**Fig. 6:**
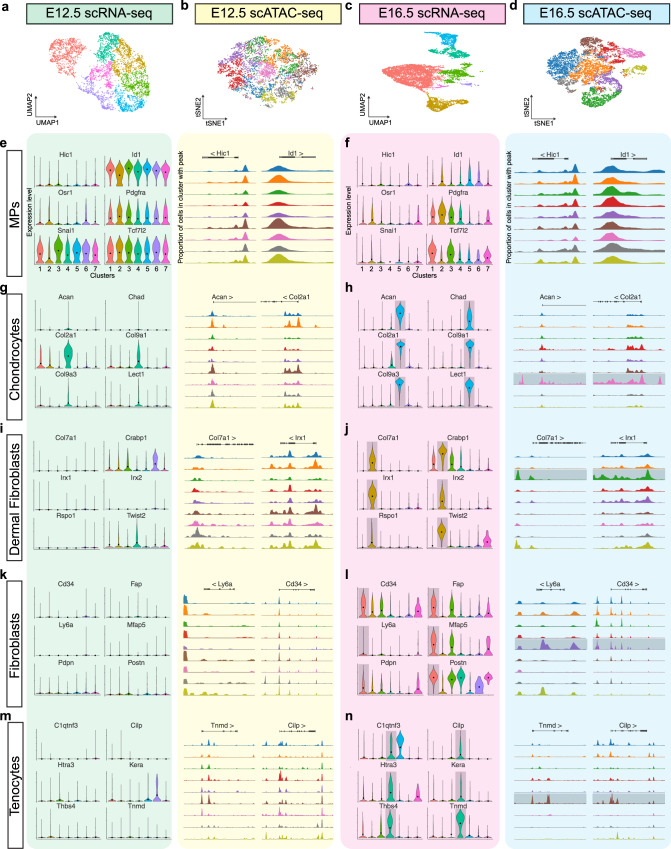
MP specification and differentiation are reflected in chromatin and transcriptome dynamics during their developmental trajectories. **a** UMAP of scRNA-seq data from E12.5-sorted limb tdTomato^+^ cells coloured according to cluster identity. **b** t-SNE plot of scATAC-seq data from E12.5-sorted tdTomato^+^ cells coloured according to cluster identity. **c** UMAP of scRNA-seq data from E16.5-sorted limb tdTomato^+^ cells coloured according to cluster identity. **d** t-SNE plot of scATAC-seq data from E16.5-sorted tdTomato^+^ cells coloured according to cluster identity. **e**, **f** Violin plots and genome tracks from representative genes for identification of MPs. **g**, **h** Violin plots and genome tracks from representative genes for identification of chondrocytes. **i**, **j** Violin plots and genome tracks from representative genes for identification of dermal fibroblasts. **k**, **l** Violin plots and genome tracks from representative genes for identification of fibroblasts. **m**, **n** Violin plots and genome tracks from representative genes for identification of tenocytes.

Comparisons between independently clustered E12.5 and E16.5 scRNA- and scATAC-seq datasets (Fig. 6a–d) revealed that genes associated with a progenitor status are widely expressed, and the respective gene loci are accessible throughout all clusters of *Hic1* MPs at E12.5 (Fig. 6e). On the other hand, expression and chromatin accessibility of these genes became restricted to specific progenitor clusters at E16.5 (Fig. 6f). Chondroprogenitors became apparent both transcriptionally and by chromatin state as early as E12.5 (Fig. 6g); this cluster became established at E16.5, with highly specific OCRs and largescale expression of chondrocyte markers such as *Acan* and *Chad* (Fig. 6h). Dermal fibroblasts (Fig. 6i, j), fibroblasts (Fig. 6k, l), and tenocytes (Fig. 6m, n) displayed a similar trend: RNA expression of cell type-specific genes were absent at E12.5 and chromatin accessibility peaks were ubiquitous throughout ATAC-seq clusters (Fig. 6i, k, m). At 16.5, cells have differentiated and this became apparent in both gene expression and chromatin landscape (Fig. 6j, l, n). Chondrocytes, tenocytes, and dermal fibroblasts were composed of similar ratios of E14.5 and E16.5 cells (Fig. 5d). To further elucidate the epigenetic signatures that characterise each differentiation trajectory, cell classification labels were transferred from the scRNA-seq dataset using an anchor-based method for cross-modality integration^47^ and individual pseudotime trajectories were constructed by subsetting the curated dataset (Supplementary Fig. 7a–f). Differential accessibility analysis in pseudotime revealed that loci associated with the chondrogenic programme remained open in the chondrocyte cluster, but become progressively more restricted in the tenocyte and DF clusters (Supplementary Fig. 7g). Similarly, OCRs within tenogenic gene loci were detected only in the tendon-enriched cluster at late pseudotime values (Supplementary Fig. 7h). Furthermore, genes reflective of a dermal fibrogenic-cell fate (*Col7a1*, *Irx1*, and *Lef1*) were only found within OCRs in the DF cluster at the late pseudotime values of the differentiation trajectory (Supplementary Fig. 7i). Collectively, these studies reveal that the early Hic1^+^ MPs represent a mesenchymal progenitor cell population capable of giving rise to various subsets of skeletal and stromal cells.

### Embryonic Hic1^+^ MP post-natal lineage contributions

**Fig. 7:**
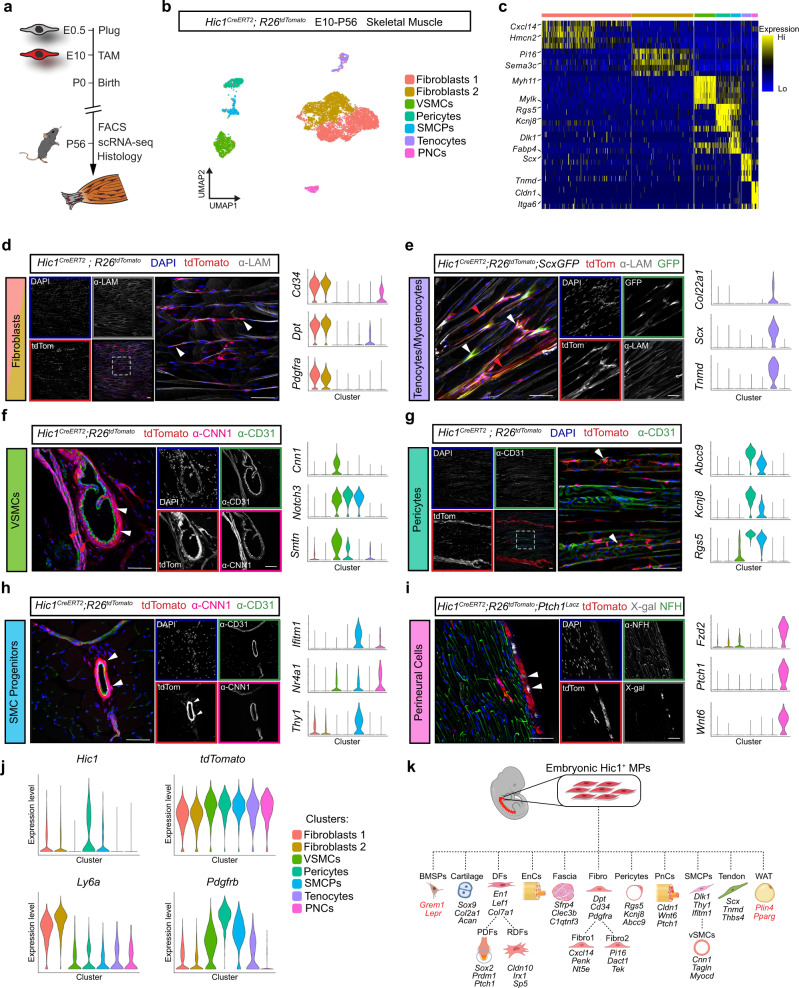
Embryonic Hic1^+^ MPs give rise to distinct populations of adult stromal cells. **a** Schematic representation of strategy employed for lineage tracing embryonic Hic1^+^ MPs into adulthood. **b** UMAP coloured by cluster of scRNA-seq data from sorted tdTomato^+^ cells isolated from the TA muscle of lineage-traced *Hic1*^*CreERT2*^*; R26*^*tdTomato*^ mice. **c** Heatmap of scRNA-seq data showing enriched genes within each cluster. **d**–**i** Representative immunofluorescence images and violin plots of each cell population identified through lineage tracing performed from E10 until P56 on *Hic1*^*CreERT2*^; *R26*^*tdTomato*^ mice. TdTomato^+^ populations persist as cells from the following lineages: fibroblasts (**d**); tenocytes and myotenocytes (**e**); pericytes (**f**); vascular smooth muscle cells (**g**); smooth muscle cell progenitors (**h**); and perineural cells (**i**). White arrowheads highlight cells of interest. White-dotted boxes highlight regions that are magnified in larger panels. *n* = 3 biologically independent mice for panels **a–i**. Scale bars, 50 µm. **j** Violin plots of select transcripts. **k** Schematic of the diverse mesenchymal limb lineages captured by labelling at E10 from development into adulthood. BMSPs, bone marrow stromal progenitors; DFs, dermal fibroblasts; EnCs, endoneural cells; Fibro, fibroblasts; PDFs, papillary dermal fibroblasts; PnCs, perineural cells; RDFs, reticular dermal fibroblasts; SMCPs, smooth muscle cell progenitors; vSMCs; vascular smooth muscle cells; WAT, white adipose tissue. Created with BioRender.com.

Hic1^+^ MP lineages emerge at various times during embryogenesis, where they contribute to a variety of mesenchymal cell types. To further interrogate Hic1^+^ MP lineage contributions, scRNA-seq was performed on skeletal muscle (SkM) samples collected at P56 (following E10 TAM administration) along with histological analyses of SkM and surrounding tissues (Fig. 7a). Bioinformatic analyses revealed 6 tdTomato^+^ populations present in SkM (Fig. 7b, c), the largest of which comprised fibroblasts (*Cd34*^*+*^*, Pdgfra*^*+*^*, Ly6a*^*+*^*, Dpt*^*+*^), which are abundant in the SkM interstitium (Fig. 7d, j). Tenocytes (*Scx*^*+*^*, Tnmd*^*+*^) and myotenocytes (*Col22a1*^*+*^) were also identified (Fig. 7e). Three clusters constituted vasculature-associated cell types (Supplementary Fig. 8a, b): Vascular smooth muscle cells (VSMCs) (*Acta2*^*+*^*, Cnn1*^*+*^*, Smtn*^*+*^) lined arteries (Fig. 7f and Supplementary Fig. 8c), pericytes (*Abcc9*^*+*^*, Kcnj8*^*+*^*, Rgs5*^*+*^) circumscribed capillaries (Fig. 7g and Supplementary Fig. 8d) and smooth muscle cell progenitors (SMCPs) (*Ifitm1*^*+*^*, Nr4a1*^*+*^*, Thy1*^*+*^) were found associated with larger blood vessels (Fig. 7h and Supplementary Fig. 8e). As expected, *Pdgfrb* was expressed to varying extents in all Hic1^+^ cell populations including *Ly6a*^*+*^ fibroblasts (Fig. 7j). Finally, perineural and endoneurial cells (*Fzd2*^*+*^*, Ptch1*^*+*^*, Wnt6*^*+*^) clustered separately in transcriptomic space and were observed in histological nerve samples (Fig. 7i and Supplementary Fig. 9a). Other tdTomato^+^ connective tissue cell types were also present in adult lineage-traced limbs, such as cortical osteocytes in the humeral shaft (Supplementary Fig. 9b), bone marrow stromal cells within the distal epiphysis of the humerus (Supplementary Fig. 9c), subcutaneous white fat throughout the limb hypodermis (Supplementary Fig. 9d), dermal fibroblasts distributed throughout the reticular and papillary dermal layers (Supplementary Fig. 9e), and mineralised fibrocartilage at the proximal head of the humerus (Supplementary Fig. 9f). Together, these analyses reveal that E10-labelled Hic1^+^ MPs contribute to select post-natal mesenchymal lineages across multiple tissues (Fig. 7k).

### Embryonic Hic1^+^ fibroblasts persist into adulthood and have roles in SkM regeneration

**Fig. 8:**
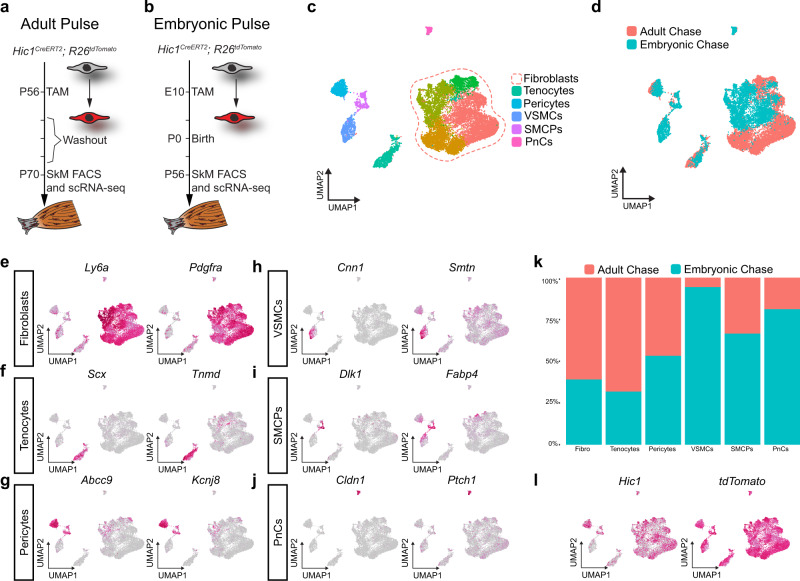
Embryonic Hic1^+^ cell derivatives persist into adulthood and display greater fate heterogeneity than adult Hic1^+^ cells. **a** Schematic of experimental timeline for lineage tracing of adult MPs. **b** Schematic of experimental timeline employed for post-natal lineage tracing of *Hic1*^*CreERT2*^; *R26*^*tdTomato*^ embryos. **c** UMAP of aggregated scRNA-seq data from adult chase and embryonic chase tdTomato^+^ MPs, coloured according to cluster ID. PnCs, perineural cells; SMCPs, smooth muscle cell progenitors; VSMCs, vascular smooth muscle cells. **d** UMAP of aggregated scRNA-seq data from adult chase and embryonic chase tdTomato^+^ MPs, coloured according to time of TAM administration. **e**–**j** UMAP plots for representative genes used to identify each cluster. **k** Stacked bar graph illustrating the composition of each cluster with respect to time of TAM dose. **l** UMAP plots for *Hic1* and tdTomato transcripts within the aggregated scRNA-seq data. Source data are provided as a Source Data file.

To determine the contribution of E10-labelled Hic1^+^ MPs to post-natal SkM homoeostasis and regeneration, scRNA-seq profiling and notexin (NTX)-induced SkM injuries were performed in adult pulse (TAM at P56) (Fig. 8a) and embryonic pulse (TAM at E10) (Fig. 8b), chase experimental models. Single-cell transcriptomic data from adult pulsed (P56–collect P70)^4^ and embryo pulsed (E10–collect P56) SkM resident tdTomato^+^ cells were aggregated, and clusters were annotated based on marker gene expression (Fig. 8c–k). Interestingly, VSMCs were only observed in the embryonic pulse-adult chase (E10-P56) experiment (Fig. 8e) and the absence of Hic1 expression in this tdTomato^+^ population reflects their more differentiated state (Fig. 8l).

**Fig. 9:**
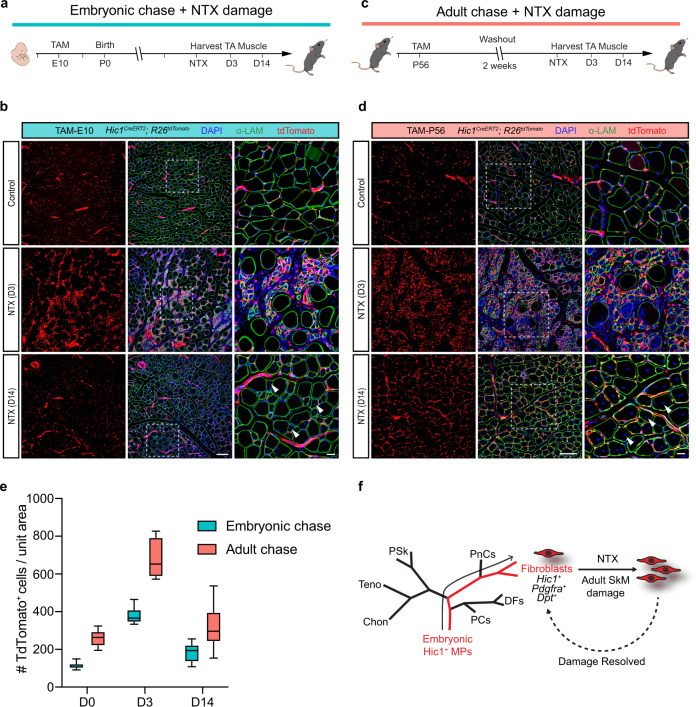
Embryonic *Hic1*-lineage fibroblasts persist post-natally and support muscle regeneration. **a** Schematic of experimental design for lineage tracing of embryonic Hic1^+^ progenitors into adulthood and subsequent muscle injury. **b** Representative *tibialis anterior* (TA) muscle sections from embryonic lineage-traced (TAM at E10) *Hic1*^*CreERT2*^; *R26*^*tdTomato*^ mice at 3- and 14-days post NTX-induced injury. Contralateral TA shown as control. *n* = 2 biologically independent animals per damage timepoint. Scale bars, 100 µm, 20 µm inset. Arrowheads indicate centrally located nuclei. **c** Schematic of experimental design for lineage tracing of adult Hic1^+^ progenitors and subsequent muscle injury. **d** Representative *tibialis anterior* (TA) muscle sections from adult lineage-traced (TAM at P56) *Hic1*^*CreERT2*^; *R26*^*tdTomato*^ mice at 3- and 14-days post NTX-induced injury. Contralateral TA shown as control. *n* = 2 biologically independent animals per damage timepoint. Scale bars, 100 µm, 20 µm inset. Arrowheads indicate centrally located nuclei. **e** Quantification of tdTomato^+^ cell abundance in embryonic and adult lineage tracing models. The box represents the interquartile range, with the centre line at the median. The whiskers extend up to 1.5 times the interquartile range (IQR). *n* = 2 or *n* ≥ 2 biologically independent animals per damage timepoint and per pulse-chase. **f** Graphical summary of the experimental results. Embryonic Hic1^+^ cells generate adult SkM-associated fibroblasts, which become activated upon injury, cell number returns to pre-injury levels following injury resolution. Source data are provided as a Source Data file.

*Hic1*^*+*^ fibroblasts emerge at ~E16.5, defined by the markers *Ly6a* and *Cd34*^7^ (Fig. 2l). Their origins and scope of contribution to embryonic development are unclear, however, studies have certainly revealed a fundamental role for this population in skeletal muscle (SkM) development^44,48^, as well as, post-natal SkM homoeostasis and regeneration^10^. Labelling at E10 yields comparable numbers of tdTomato^+^ cells (SkM-associated fibroblasts) to labelling at P56 (Fig. 8d, e), revealing that Hic1^+^ MPs are a major source of post-natal fibroblasts including the two universal fibroblast populations, *Dpt*^+^ *Pi16*^+^ and *Dpt*^+^ *Col15a1*^+^^4,11^. Furthermore, consistent with prior findings^4,6,7,9^, notexin (NTX)-induced SkM injury leads to a marked expansion of fibroblasts (Fig. 9a, b), which is comparable to that of Hic1^+^ cells labelled at P56 (Fig. 9c, d). Overall, the labelled cells display a similar post-damage proliferative phase followed by a return to baseline numbers (Fig. 9e). These findings indicate that embryonic Hic1^+^ MPs give rise to fibroblasts, which persist into adulthood and contribute to muscle regeneration (Fig. 9f).

## Discussion

In adult skeletal muscle, *Hic1* identifies three major MP subpopulations including, fibroblasts, pericytes and tenogenic progenitors. Herein, *Hic1*-based genetic lineage tracing tools have been used to develop an atlas of Hic1^+^ MP derivatives starting from the onset of *Hic1* expression in limb mesenchyme through to P56. In this regard, *Hic1* defines a rare sub-population of cells in the early limb mesenchyme. While barely evident at E11.5, Hic1-lineage-traced cells subsequently undergo appreciable expansion, culminating in embryonic and post-natal progeny that reflect the diversity of fibroblasts, other specialised MPs and mural cells. A number of other markers have been used to identify various subpopulations within these lineages including *Grem1*^49^, *Lepr*^50^, *Osr1*^44^, and *Tcf7l2*^8^, but *Hic1* appears to identify an MP compartment(s) situated at the apex of this lineage tree, thereby capturing the totality of their fates. With the exception of VSMCs, there is excellent concordance between the embryonic lineage-traced derivatives and those identified in the adult. Furthermore, again with the exception of VSMCs, the embryo-traced Hic1^+^ derivatives express *Hic1* in the adult, indicating that their progenitor status is likely maintained.

In the URD-generated mesenchymal lineage tree, *Hic1* is expressed in the trunk and branch populations and is lost in specific tip populations associated with the emergence of differentiated phenotypes, i.e., chondrocytes and tenocytes. *Osr1* was previously shown to define embryonic fibroblasts and its expression parallels that of *Hic1*, except it is downregulated in the emerging pericyte lineage as well as in adult SkM pericytes. Similarly, *Pdgfra* is a useful marker for identifying various MPs, however, it is also absent in pericytes and is accordingly downregulated in the pericyte branch. *Dpt* is expressed much later in embryonic development along with *Ly6a* and defines the emergence of the Fibro1 and 2 populations. *Hic1* effectively labels these two major emerging mesenchymal cell branches, fibrogenic-skeletogenic and pericytic within the developing limb. Collectively, these findings appear to indicate two separate but major branches defining the embryonic limb mesenchyme, a fibrogenic-skeletogenic branch and a distinct pericyte branch.

### Hic1^+^ progeny contribute to fibroblast and peripheral nerve mesenchymal components

A recent study has defined two “universal” fibroblast populations that are shared across tissues, defined by the transcripts *Dpt*, *Col15a1* (Fibro1) and *Dpt, Pi16* (Fibro2)^11^. The URD plots identify profiles reflective of these two populations and while *Col15a1* is not expressed in the Fibro1 compartment at E16.5, other genes associated with this sub-population (*Cxcl12*, *Fbln1, Penk*) were found to be enriched in this branch. A recent study that employed scRNA-seq to characterise EGFP^+^ cells from a *Pdgfrb*^EGFP^ knock-in, further refined the classification of SkM fibroblast types and their contribution to the muscle connective tissues^51^. They identified presumptive signatures reflective of endomysial cells, perimysial and epimysial cells and a new population they refer to as paramysial cells, which are situated between the endomysium and perimysium. Interestingly, the Dpt^+^ Pi16^+^ (Pdgfra^+^ Hic1^+^) sub-population was enriched in the endomysium, which is a thin connective tissue layer that circumscribes the individual myofibres. This population selectively expresses transcripts of numerous trophic factors (i.e., *Wnt2*, *Wnt5a*, *Sema3c*), which is consistent both with the proximity of the endomysium to the satellite cell niche and its function in regulating this compartment.

A previous study identified multiple Hic1^+^ MP types within the adult dermis, including: hair follicle fibroblast progenitors (*Sox2*^*+*^, *Itga8*^*+*^, *Mgp*^*+*^, *Acan*^*+*^), which repopulate the dermal sheath (*Itga8*^*+*^*, Itga5*^*+*^*, Acan*^*+*^*, Myh10*^*+*^*, Myl9*^*+*^*, Cd200*^*+*^) and dermal papillae (*Sox2*^*+*^*, Lef1*^*+*^*, Crabp1*^*+*^*, Rspo3*^*+*^*, Corin*^*+*^*, Vcan*^*+*^) fibroblast compartments, papillary fibroblasts (*Dpp4*^*+*^*, Dlk1*^*-*^*, Blimp1*^*+*^*, Lrig1*^*+*^*, Ly6a*^*-*^*, Trps1*^*+*^), and reticular fibroblasts (*Dpp4*^*-*^*, Dlk1*^*+*^*, Ly6a*^*-*^)^13^. During embryogenesis these populations emerge from a common (*Pdgfra*^*+*^*, Dlk1*^*+*^*, Lrig1*^*+*^) fibroblast progenitor^52^. *Pdgfra* and *Dlk1* are broadly expressed throughout many of the branches, whereas *Lrig1*^+^ cells are restricted to the presumptive DF lineage. This parallels the appearance of *En1*, another DF progenitor marker. At 14.5, the DF lineage bifurcates into PDFP and RDFP branches reflected by the differential expression of *Blimp1*, *Lef1*, *Vcam1*, and *Trps1*. At this branch point, *Sox2* transcripts are detectable in, and restricted to, the PDFP branch and expression can be further confirmed by anti-SOX2 staining at E18.5. Consistent with previous reports, the PDFP branch will give rise to all of the specialised fibroblasts within the skin and signatures reflective of this potential are embedded in this branch tip, including: *Cd200*, *Col11a1*, *Lef1*, *Myl9*, and *Rspo3*. Furthermore, the point of RDFP and PDFP bifurcation is associated with differential expression of multiple *Irx* genes, with *Irx1*, *3* and *5* enriched in the RF branch. In addition to *Sox2*, other Sox transcripts are preferentially expressed in the PDFPs, such as *Sox11* and *Sox18*. In comparison to all of the other lineages, the dermal fibroblast lineage exhibits a prominent canonical WNT signature that is associated with *Axin2*, *Lef1* and *Nkd2* expression immediately following the emergence of this lineage at E12.5. Collectively, *Hic1* effectively labels the common fibroblast progenitor in the E12.5 limb mesenchyme and continues to be expressed in these lineages, identifying enduring progenitor subsets, which support skin homoeostasis and regeneration into and throughout adulthood^13^.

In a simple ex vivo culture experiment, Bunge et al. showed that the perineurium appeared to derive from fibroblasts^53^. *Hic1* progeny give rise to all of the nerve-associated fibroblasts and in the adult, these cells continue to express *Hic1*, indicative of their progenitor status. In the URD lineage tree presented here, cells reflective of the perineurium branch off from the common Dpt^+^ fibroblast branch and markers reflective of a perineurial signature (*Wnt6*, *Ptch1*, *Cldn1*) can be detected at E16.5. Hic1^+^ cells traced from E10 to P56 clearly show contribution to the various mesenchymal components of the sciatic nerve, including the epineurium, perineurium and endoneurium. The signatures of the perineurium *(Wnt6, Cldn1, Ptch1)* and endoneurium (*Sox9*, *Cd34*, *Fzd2*) align well with the recently published scRNA-seq sciatic nerve dataset (SNAT)^36^. However, the epineurial cluster within this data aligns with the *Dpt*^*+*^ fibroblast cluster and as such, is likely embedded within the Hic1^+^ fibroblast clusters.

### Hic1^+^ MPs and “classic” mesenchymal cell types

Mesenchymal cells are, in part, functionally defined by their ability to contribute to the skeletogenic lineages, including, chondrogenic, osteogenic and/or tenogenic. Strong transcriptomic signatures reflective of tenogenic and chondro/osteogenic fates are evident in the URD plots and were validated with histological analyses. In addition, within the tenogenic branch, a *Col22a1-*containing tenogenic signature could be identified reflective of myotenocytes within the myotendinous junction^54,55^. Within embryonic and adult limb bones, lineage-traced osteocytes as well as presumptive bone marrow stromal cells could be identified based on their morphology. BMSCs and osteocytes were located in cortical and trabecular bone of the humerus and corresponding bone marrow, respectively. Neither of these populations could be reliably detected in the embryonic URD plots. An osteogenic signature is embedded in the tip of the chondrogenic branch and this could reflect a bona fide osteogenic lineage and/or chondrocyte maturation and the appearance of hypertrophic chondrocytes (HC), which express many osteoblastic markers. Consistent with this, the tip population is also positive for the HC-expressed gene *Col10a1*. Alternatively, Hic1 progeny make a limited contribution to embryonic bone formation and thus, the detection of this population would be compromised by its rarity and/or difficulty in liberating tdTomato^+^ cells from bone. In this regard, this population is remarkably similar to the Lepr^+^ bone mesenchymal stromal cell^50^. In the post-natal bone marrow, *Lepr* identifies the majority of the mesenchymal stromal population. While Lepr^+^ cells can be identified in the embryonic bone marrow, this population makes a negligible contribution to osteocytes therein^50^. In contrast, Lepr^+^ cells contribute substantially to new osteocytes generated with normal adult bone turnover and following fracture^50,56^. Very few tdTomato^+^ cells can be identified in the embryonic cortical bone and thus, it appears that *Hic1* progeny make a limited contribution to the embryonic skeleton.

Multipotent MPs are, in part, defined by their ability to generate white adipocytes. Transcripts indicative of adipogenic lineage potential (*Cebpa*, *Cebpb*, *Cebpd*, *Pparg*) were embedded in multiple branches (including fibro 1, fibro 2, perineurium, dermal fibroblasts, and fascia), however, they were most prevalent within the fibroblast 1 and 2 branches. While tdTomato^+^ PLIN1^+^ adipocytes were detected histologically in the post-natal limb, this population was not detected in scRNA-seq. This likely reflects the technical challenges in sorting large fragile adipocytes and their subsequent inefficient capture into droplets in drop-seq-based methods.

### Hic1^+^ MPs represent a source of mural cells

Pericytes form a distinct lineage within the URD tree and this is reflected by their expression of a core group of transcripts, which exhibit limited overlap with fibroblasts. Within the time points captured, pericytes appear after the branch point at E12.5 and are defined by the transcripts *Rgs5*, *Kcnj8*, *Abcc9*, *Myh11* with *Myocd* appearing weakly in the tip cells. Interestingly there are a number of transcripts broadly expressed at the top of the tree that are quickly extinguished in the pericyte arm including *Osr1, Pdgfra, Scx, Tcf7l2, Sox11, Snai1*, and *Crabp2*. The transcriptional programme underlying the specification of the pericytic lineage has yet to be identified^38^. However, these findings would indicate that the acquisition of a pericytic fate may be, in part, related to the decreased expression of transcription factors and/or signalling pathways associated with alternative fates. Unexpectedly, the E10-P56 chase revealed a major tdTomato^+^ *Hic1*^*-*^ population of VSMCs (*Cnn1*^+^, *Tagln*^+^, *Myocd*^+^, *Myh11*^*+*^, and *Pdlim3*^*+*^) and tdTomato^+^ VSMCs (CNN1^+^) were detected in the tunica media of arterioles within SkM. A distinct hybrid cluster expressing transcripts reflective of VSMCs and pericytes was also detected. However, in addition to many VSMC and pericytic markers, this cluster also expresses several unique transcripts including *Dlk1*, *Adra2a*, *Rgs16* and is abundant for *Cd90*. *Dlk1* is typically associated with stem and progenitor cells and a similar *Dlk1*^+^ *Thy1*^+^ population has been described in human subcutaneous fat, located within the vessel adventitia and on capillaries^57^. Furthermore, *Dlk1* expression has been defined in a subset of brain pericytes^58^. Thus, the *Hic1*^+^ *Dlk*^+^ population appears to represent a distinct pericyte-like progenitor along a pericyte to VSMC lineage continuum^51,59^.

### Hic1^+^ limb MPs have a somitic origin

Hic1^+^ MPs represent a distinct mesenchymal population, which emerges later in limb development, after the initiation of the LPM-derived primary skeletal elements. This MP sub-population appears coincident with migrating somite-derived myogenic precursors and infiltrating neuronal processes. Nonetheless, Hic1^+^ MPs generate a unique cellular lineage hierarchy, which culminates in embryonic and post-natal progeny that reflect the diversity of specialised functions of MPs. While the limb contains a variety of cell types that have a somitic origin and derive from the sclerotome and myotome^21,22,28^, the embryonic origin of many of these mesenchymal lineages is unclear. Early chick-quail transplant experiments have indicated a somitic origin for VSMCs^28^, whereas most of the other mesenchymal lineages appear to derive from the LPM. Furthermore, retrovirally marked clonal tracing performed in the early chick limb revealed that a population of early progenitors with multi-lineage potential contributed to up to five distinct lineages including, chondrocytes, perichondrial cells, tenocytes, SkM connective tissue cells (fibroblasts) and dermal fibroblasts^60^. An additional somitic structure, the syndetome, has been largely considered to be restricted to the formation of axial tendons^61^. Here, we propose that Hic1^+^ MPs identified at E10 within the syndetome and sclerotome compartments of the somitic mesoderm, represent a migratory cell population that invades the developing limb bud and ramifies into diverse stromal and skeletogenic fates. With the use of a new genetic lineage tracing model involving labelling of Hic1^+^ cells, we are able to identify a subset of Scx^+^ cells within the syndetome with a hitherto undescribed contribution to appendicular development. In this regard, Hic1^+^ progenitors within the syndetome extend into the hypaxial edge of the somite where they are in close contact with the Pax7^+^ myotome. At this site, *Hic1* lineage-traced cells migrate into the E11.5 limb bud in close proximity with PAX3^+^ myogenic progenitors and invading nerve axons. Myogenic progenitors initiate their migration at E10.5^62^ and in agreement with this, we observed PAX3^+^ cells within the limb bud at E11.5 suggesting that Hic1^+^ MP migration represents a secondary wave of progenitors entering the limb bud.

The limited complexity observed in the single-cell-omics analyses of tdTomato^+^ cells at E11.5 and E12.5, is consistent with a common embryonic origin for the Hic1^+^ MPs. This is further supported by scATAC-seq analyses where OCRs are identified in a single cluster for multiple lineages, such as *Scx*, *Sox9*, and *Irx1* for the tenogenic, chondrogenic and, dermal fibroblast lineages, respectively. Analysis of the respective scATAC-seq pseudotime trajectories, indicates that with lineage progression and cell commitment, these alternative fates and associated OCRs are lost. These comments notwithstanding, these studies do not preclude the possibility of multiple overlapping populations with more restricted lineage trajectories. The potentially distinct lineage trajectories of fibrogenic/skeletogenic arms versus the pericyte/VSMC branch in part reflect this dichotomous origin. Alternatively, *Hic1* may represent a functional marker that is expressed in disparate mesenchymal lineages, indicative of a shared regulatory programme.

### Embryonic Hic1^+^ MP progeny in adult tissue renewal and regeneration

Adult tissues harbour a variety of resident MP populations that serve vital functions in tissue homoeostasis, growth, renewal and regeneration. The origin of many of these populations has remained nebulous. Tracing of E10-labelled Hic1^+^ MPs demonstrated a contribution to numerous terminally differentiated embryonic mesenchymal cell types, but is also sufficient to capture many of these post-natal MP populations. In particular, post-natal SkM interstitial fibroblasts are efficiently labelled at E10, and congruent with known adult fibroblast/FAP activity^4,6,7,10^, undergo a notable expansion following injury and during regeneration. Adult Hic1^+^ MPs figure prominently in these processes^4,6,10^, indicating that embryonic Hic1^+^ MPs not only support limb ontogenesis, but also persist and serve as a reservoir for post-natal tissue maintenance and restoration.

## Methods

### Mice

Animals were maintained and experimental protocols were conducted in accordance with approved and ethical treatment standards of the Animal Care Committee at the University of British Columbia.

The generation of *Hic1*^*nLacZ*^ and *Hic1*^*CreERT2*^ mice have been previously described^4^. Other mouse lines include: B6.Cg-Gt(ROSA)26Sor^tm14(CAG-tdTomato)Hze^/J (Jax stock number 007914; herein referred to as *Rosa*^Tom^), C57BL/6 J (Jax stock number 000664), *ScxGFP*^63^, Crl:CD1(ICR) (Charles River strain code 022), B6.129S4-*Pdgfra*^*tm11(EGFP)Sor*^/J (Jax stock number 007669), and *Ptch1*^*tm1Mps*^*/J* (Jax stock number 003081).

For lineage tracing experiments, *Hic1*^*CreERT2*^ mice were interbred with *ROSA26*^*Tomato*^ mice to generate *Hic1*^*CreERT2/CreERT2*^; *ROSA26*^*Tom/Tom*^ progeny, *Hic1*^*CrERT2*^; *R26*^*tdTomato*^. To induce CreERT2 nuclear translocation, timed-pregnant females were administered a single dose of TAM at a concentration of 300 mg/kg in sunflower oil via oral gavage. Unless otherwise stated, TAM was administered the night of embryonic day 10.0 for all experiments. For lineage tracing *Hic1*^*CreERT2*^; *R26*^*tdTomato*^ cell progeny into adulthood, timed-pregnant females were administered a single dose of TAM at a concentration of 300 mg/kg along with 150 mg/kg progesterone (Sigma P3972-5G) in sunflower oil via oral gavage. The morning of E20.5, the mother was sacrificed and fetuses were collected via caesarean section and cross-fostered with a CD1 litter. For fate mapping involving the tendon compartment, *Hic1*^*CreERT2*^; *R26*^*tdTomato*^ mice were interbred with *ScxGFP* homozygous mice to generate *Hic1*^*CreERT2*^; *R26*^*tdTomato*^; *ScxGFP* mice. These mice were outbred to a CD-1 background for five generations. Mice were housed under standard conditions (12 h light/dark cycle) and provided food and water *ad libitum*. All experiments involving use of embryonic tissues consisted of male and female embryos analysed indiscriminately.

### Muscle injury

Muscle injury of the tibialis anterior muscles were induced by intramuscular injection of 0.2 µg of notexin (NTX) snake venom extract (Latoxan) in 20 µL PBS (10 µg/mL)^7^ on 8–10-week-old *Hic1*^*CrERT2*^; *R26*^*tdTomato*^ males. Muscles were harvested four days post injury and processed for immunofluorescence staining as described below. *n* = 3 mice per timepoint for adult lineage tracing. For embryonic lineage tracing *n* = 3 mice for Day 3; and *n* = 2 for Day 14. Contralateral TAs were used as controls.

Quantification of linage-traced tdTomato^+^ cell expansion after NTX-induced muscle injury was carried as follows: Six representative images were taken from three injury timepoints (day 0, day 3, and day 14). tdTomato^+^ cells were enumerated using the ImageJ count function ensuring localisation with DAPI to identify bona fide cells. Results were tabulated and plotted as the mean ± standard deviation.

### Immunofluorescence staining

Embryos were collected at indicated time points and fixed in 2% PFA for 72 h. Following fixation, samples were cryoprotected by processing through increasing concentrations of sucrose up to 50%. Samples were subsequently embedded in Optimal Cutting Temperature (OCT–Tissue Tek 4583) embedding medium by use of an isopentane cold bath within liquid nitrogen. Histological cryosections were prepared at 20 μm on Superfrost Plus slides (VWR 48311-703) unless otherwise stated. Slides were incubated in sodium borohydride (10 mg/mL in PBS) for 45 min to reduce autofluorescence. Following PBS washes, slides were incubated with blocking solution [2.5% goat serum (Gemini 100–190); 2.5% BSA (Sigma A7030)] in PBS for 1 h. Primary antibodies were diluted in blocking solution at appropriate concentrations and incubated on slides overnight at 4^o^C. Secondary antibodies were diluted similarly and incubated for 45 min at room temperature. All slides were counterstained with DAPI (600 nM) and mounted with Aqua Polymount (Polysciences 18606). The following antibodies were used for the identification of cells and tissues in embryonic forelimbs: α-Myosin heavy chain, sarcomere, clone: P3U-1, Cat. #MF 20, Lot: 7/6/17, DSHB, 1:50; α-MYOD1, clone: EPR6653-131, Cat. #ab133627, Lot: GR3375193-2, Abcam, 1:100; α-PAX1, clone: 5A2, Cat. #ab252847, Lot: GR3345842-1, Abcam, 1:100; α-SOX10, clone: EPR4067, Cat. #ab155279, Lot: GR113617-36, Abcam, 1:100; α-SOX9, clone: EPR14335-78, Cat. #ab185966, Lot: GR3241181-3, Abcam, 1:100; α-CD31, clone: MEC 13.3, Cat. #550274, Lot: 8079850, BD Pharmingen, 1:50; α-myosin (skeletal, fast), clone: MY-32, Cat. #M4276, Lot: 105M4841V, Sigma Aldrich, 1:1000; α-PAX7, clone: PAX-7, Cat. AB0000456, UBC Ablab, 1:200; Anti-Neurofilament heavy chain, Cat. #ab4680, Lot: GR3241438-11, Abcam, 1:5000; α-RFP, Cat. #ab62341, Lot: GR3184770-3, Abcam, 1:100; Anti-GFP, Cat. #ab13970, Lot. GR236651-21, Abcam, 1:100; α-COL22A1, Cat. #ab121846, Lot. GR223910-6, Abcam, 1:100. For detection of HIC1 with α-HIC1 (C25; 1:2000 dilution) a tyramide staining strategy was used according to the manufacturer’s instructions (ThermoFisher T20926). Secondary antibodies were all used at a concentration of 1:500 and consisted in Alexa Fluor 488 goat anti-mouse IgG, Cat. #A11029, Lot: 1252783, Thermo Fisher Scientific; Alexa Fluor 647 goat anti-mouse IgG, Cat. #A32728, Lot: VH311610, Thermo Fisher Scientific; Alexa Fluor 647 goat anti-rat IgG, Cat. #A21247, Lot: 2089926, Thermo Fisher Scientific; Alexa Fluor 594 goat anti-rabbit IgG, Cat. #A11012, Lot: 2018240, Thermo Fisher Scientific; Alexa Fluor 488 goat anti-rabbit IgG, Cat. #A11034, Lot: 2018207, Thermo Fisher Scientific; Alexa Fluor 647 goat anti-rabbit IgG, Cat. #A27040, Lot: RB232998A, Thermo Fisher Scientific; Alexa Fluor 488 goat anti-rat IgG, Cat. #A11006, Lot: 2048174, Thermo Fisher Scientific; Alexa Fluor 488 goat anti-chicken IgG, Cat. #A11039, Lot: 1937504, Thermo Fisher Scientific.

Whole-mount visualisation of *Hic1*^*CreERT2*^; *R26*^*tdTomato*^ and *Hic1*^*CreERT2*^; *R26*^*tdTomato*^; *ScxGFP* embryos was achieved by clearing samples using the CUBIC tissue clearing protocol^64^. An organic BABB-based staining and clearing technique^65^ was employed for whole-mount immunofluorescence analyses of *Hic1*^*CreERT2*^; *R26*^*tdTomato*^ embryos.

To minimise erythrocyte-derived autofluorescence on *Hic1*^*CreERT2*^; *R26*^*tdTomato*^ tissue sections in Fig. 1c, Vector TrueVIEW autofluorescence quenching kit (SP-8400) was used as per the manufacturer’s instructions.

Staining of embryonic cryo-embedded samples with α-PAX3 (clone: C2, Cat. #Pax3, DSHB, 1:50) required a compromise between maintaining tissue integrity and maximising fluorescence signal. Antigen retrieval in 10 mM citrate buffer (pH 6.0) at 60° C overnight (>12 h) allowed for epitope exposure with minimal impact on the quality of the tissue section. After antigen retrieval, slides were rinsed 3x in PBS and the immunofluorescence staining procedure was then followed as described above with one exception, NaBH_4_ quenching was not performed on slides that were exposed to α-PAX3 as this compromises signal detection. When combining α-PAX3 staining with the α-HIC1 tyramide amplification protocol a four-day protocol is required to achieve signal detection. On day 1, antigen retrieval for the PAX3 epitope was carried out overnight. On Day 2, the immunofluorescence protocol (ensure NaBH_4_ step is omitted) was initiated and incubation with α-PAX3 was performed overnight at 4 °C. Incubation in primary antibody overnight is paramount to achieve a strong signal. Day 3 involves completion of the α-PAX3 staining with an appropriate secondary antibody followed by 3x PBS washes and then incubating in α-HIC1 antibody overnight. Again, overnight incubation was required for to achieve a detectable signal. On Day 4, the final step involves completing the tyramide signal amplification protocol as described above. Immunofluorescence data were collected using CellSens Dimension 1.18 and NIS-Elements 4.60.00, brightfield whole-mount images were collected using Q-capture Pro 7.0.30 software.

### Quantitative PCR on embryonic forelimbs

RNA was isolated from embryonic forelimbs using Trizol (Invitrogen). The quality and concentration of isolated RNA was determined using a NanoDrop ND-1000 spectrophotometer. cDNA was generated using a High Capacity cDNA Reverse Transcription kit (Applied Biosystems) as per manufacturer’s instructions. Approximately 2 μg or the maximum amount of RNA available was transcribed in a 20 μL reaction volume. To follow the expression of *Hic1* gene transcripts, quantitative real-time PCR was carried out using the 7500 Fast Real-Time PCR System (Applied Biosystems) with the following primer/probe combinations: forward-CGGTGGCAAGTTTGCTCAA, Reverse-CTACAGCGTGCTCTTCATATGG, Probe- AGCGCAACCTCATC.

### Whole-mount and cryosection X-gal staining

β-galactosidase enzymatic activity at different stages of embryonic limb development was detected using established whole-mount X-gal staining protocols. Embryos were collected at various developmental time points ranging from E11.0 to E14.5. Dissected limbs were fixed in LacZ fixative (100 mM MgCl_2_, 0.2% glutaraldehyde, 5 mM ethylene glycol tetra-acetic acid in 2% PFA). Duration of immersion was dependent on the timepoint collected: E11.0 and E11.5 limbs were fixed for 1 h, E12.5 for 1.5 h, E13.5 for 2 h, and E14.5 for 2.5 h. All fixative incubation periods were carried out in the dark at 4 ^o^C. Fixed samples were washed 3x in cold PBS for 30 min each followed by overnight incubation in LacZ staining solution (2 mM MgCl_2_, 0.01 % Deoxycholate, 0.02% NP40, 5 mM potassium ferricyanide, 5 mM potassium ferrocyanide and 1 mg/mL 5-bromo-4-chloro-3-indolyl-β-D-galactopyranoside) at 37 ^o^C. Samples were post-fixed in 4% PFA for an hour at 4 ^o^C prior to imaging.

To generate X-gal-stained histological embryonic limb sections, pregnant mice were sacrificed and embryos were isolated in cold PBS. Limbs were subsequently dissected from the trunk and fixed in LacZ fixative as described above. Fixed limbs were washed three times and processed for cryosectioning as described above. Samples were subsequently processed for X-gal staining by cryosectioning the frozen OCT blocks with a Leica CM 1950 cryostat at 6–10 μm. For staining, slides were thawed at room temperature and then washed 3 × 10 min in PBS to remove residual OCT compound followed by incubation overnight in LacZ staining solution (see previous section) at 37 ^o^C. Once stained, slides were washed in PBS 3 × 5 min, counterstained with Nuclear Fast Red (NFR) for 2 min, rinsed in water and dehydrated through a series of solutions as follows: 2 × 2 min in 70% ethanol, 2 × 2 min in 95% ethanol, 2 × 5 min in 100% ethanol, and 2 × 10 min in xylene. Slides were then cover-slipped with Cytoseal 60 mounting media.

### Flow cytometry and fluorescence-activated cell sorting (FACS) methodology

Freshly dissected E11.5 and E12.5 *Hic1*^*CrERT2*^; *R26*^*tdTomato*^ or *Hic1*^*CreERT2*^; *R26*^*tdTomato*^; *ScxGFP* embryo forelimbs were digested in a Dispase enzyme-based solution [Dispase II 10 units/mL (Roche 4942078001); 10% fetal bovine serum in Puck’s saline A]. Two-three forelimb pairs were pooled into 3 mL reactions. For E13.5, E14.5 and E16.5 forelimbs, the dissociation cocktail consisted of Collagenase A (Roche, 10103586001; 1.5 mg/mL), Pronase (Roche, 10165921001; 0.5 mg/mL), DNase (Roche, 11284932001; 100μg) in Dulbecco’s Modified Eagle Medium (DMEM; Gibco, 11960-044). Single-forelimb pairs were dissociated in 2 mL reactions. In both cases, enzymatic digestion was carried out at 37 °C with gentle rotation for 1.5 h with vortexing every 30 min. Digested material was then triturated by pipetting and passed through a 40 μm cell strainer (Falcon, 352350). The resultant cell suspension was washed with FACS buffer (2 mM EDTA, 2% FBS in PBS) and centrifuged at 500 x *g* for 5 min. To further enrich for MPs, an antibody panel in combination with tdTomato fluorescence, was designed to label and exclude lineage-positive cells [anti-CD45-647 (UBC Ablab 67-0047-01, 1:400), anti-CD117-APC (eBiosciences 17-1172-82, 1:500), anti-CD31-APC (BD Biosciences, 551262, 1:400), anti-CD11b-647 (UBC Ablab 67-0055-01, 1:500), anti-F4/80-647 (UBC Ablab, 67-0035-05, 1:500), and anti-Ter119-647 (UBC-Ablab, 67-0031-01, 1:200)]. Cells were incubated in staining cocktail for 30 min on ice in the dark. Subsequently, cells were washed in 15 mL FACS buffer and centrifuged at 500 x *g* for 5 min. The cell pellet was resuspended in FACS buffer containing Hoechst 33342 (Sigma B2261; final concentration 4 mM) and propidium iodide (Thermofisher, P1304MP; 1 mg/mL). Cells were enriched using a BD Influx cell sorter and a combination of Hoechst, forward/side scatter parameters and propidium iodide were used to identify viable cells. Sorted cells were collected into sort media (20% FBS, l-glutamine (2 mM), penicillin (100 units/mL), and streptomycin (100 μg/mL in DMEM) in cooled siliconized microcentrifuge tubes (Fisher Scientific; 02-681-320). Flow cytometry data were collected using BD FACS Diva. Cells were sorted using BC FACS Sortware. FlowJo was used to analyse and visualise all cytometer and FACS data.

### Cell cycle analysis

To detect proliferating cells in embryonic forelimbs, standard methodology involving analysis of the incorporation of the nucleoside triphosphate analogue 5-ethynyl-2’-deoxyuridine (EdU) was used. For this purpose, pregnant dams were injected intraperitoneally with a single dose of 1.5 mg of EdU (ThermoFisher E10415) in PBS. Embryos were collected 24 h post-injection and forelimb buds were processed for histology or flow cytometry using the Click-iT Plus EdU Alexa Fluor 647 Imaging kit (ThermoFisher C10640) and the Click-iT Plus EdU Pacific Blue Flow Cytometry Assay kit (ThermoFisher C10636), respectively, both as per manufacturer instructions. *n* = 3 independent litters per timepoint tested.

### Single-cell RNA-Seq

Forelimb pairs were collected, pooled and processed from *Hic1*^*CreERT2*^; *R26*^*tdTomato*^ litters at five different developmental timepoints with the following numbers at each timepoint: 14 (E11.5); 7 (E12.5); 6 (E13.5); and 6 (E14.5). At E16.5, forelimb pairs were divided into proximal and distal samples. The proximal limb segments consisted of 6 forelimb segments containing only the distal stylopod, proximal zeugopod and their articulation (elbow joint). The distal counterpart consisted in the distal zeugopod and autopod. For E11.5 whole 1 and 2 samples, whole *Hic1*^*CreERT2*^; *R26*^*tdTomato*^ limbs were dissociated and total lineage-negative cells captured. Single-cell suspensions were generated and tdTomato^+^ cells were enriched by FACS (see above) into 0.22 μm vacuum filtered SC collection media (DMEM containing 5% FBS). Cells were counted and quality control was evaluated by hemocytometer. If the single-cell suspension was confirmed to contain >98% tdTomato^+^ cells, the solution was input into a Chromium Controller (10x Genomics). Cell capture and library generation was carried out with the Chromium single-cell 3’ reagent kit v2 (10x Genomics) for E11.5, E12.5, and E13.5 samples and v3 for E14.5 and E16.5. cDNA libraries were sequenced on a Nextseq500 (Illumina) to a depth >40,000 reads per cell. The generated reads were aligned to a modified mm10 reference genome^4^. Demultiplexing and sequence alignment was performed using the cellranger *count* pipeline (10x Genomics). Quality metrics for the scRNA-seq were obtained from the cellranger-*count* output (Supplementary Data 1). Aggregated library sets were generated using the cellranger *aggr* pipeline. Principal component analysis, non-linear dimensionality reduction (t-SNE and UMAP), and clustering were done using the Monocle3 R-package^66,67^. To ensure only high-quality cells were integrated into further bioinformatic analyses, cells with low total UMI count (<3000) and high mitochondrial RNA content were filtered. Also, the E16.5 forelimb datasets contained small clusters of contaminating epithelial cells (*Krt1*^*+*^ or *Krt14*^*+*^), these cells were filtered from the aggregated dataset. A parallel set of principal component and downstream analyses were performed using the Seurat R package^68^. Monocle3 clustering results were subset into the Seurat object to generate violin plots and heatmaps.

For scRNA-seq of MPs from adult muscle, single-cell suspensions were generated as previously described^4^. Briefly, TA muscles pooled from two P56 *Hic1*^*CreERT2*^*; R26*^*tdTomato*^ hindlimbs and were sequentially digested in a volume of 250 µL per TA containing 500 U/mL Collagenase II (Sigma C-6685-5G) for 30 min followed by a cocktail of 1.5 U/mL Collagenase D (Roche 11088882001) and 2.4 U/mL Dispase II (Roche 04942078001) for 60 min. Digested material was subsequently stained and sorted as described above with the following modification: After the initial sort, viable target cells were further purified by sorting second time. If the single-cell suspension was confirmed to contain >98% tdTomato^+^ cells, the solution was input into a Chromium Controller (10x Genomics). Cell capture and library generation was carried out with the Chromium single-cell 3’ reagent kit v3. cDNA libraries were sequenced on a Nextseq500 (Illumina) to a depth >50,000 reads per cell. The generated reads were aligned to a modified mm10 reference genome^4^. Demultiplexing and sequence alignment was performed using the cellranger *count* pipeline (10x Genomics). Quality metrics for the scRNA-seq were obtained from the cellranger-*count* output (Supplementary Data 1). Cells containing low total UMI counts (<1500), high mitochondrial RNA content and small clusters containing cycling cells (*Birc5*^*+*^, 90 cells), and contaminating cell types (*Mpz*^*+*^, 146 cells; *Pecam1*^*+*^, 19 cells) were removed from further downstream analyses. Principal component analysis, non-linear dimensionality reduction (t-SNE and UMAP), and clustering were done using the Seurat R package^68^.

### Single-cell fate trajectory reconstruction

The URD R package which involves a simulated diffusion-based approach was used to computationally generate differentiation trajectories in pseudotime at the single-cell level^31^. Cells undergoing mitosis confounded analysis and were removed from subsequent analyses. Transition probabilities and pseudotime were calculated as per the URD tutorial (https://github.com/farrellja/URD). Cells originating from E11.5 forelimbs were assigned as the root. Tip populations were determined manually through analysis of differentially expressed marker genes in the E16.5 dataset (fid. S2B-C; Supplementary Data 2 includes the top 500 enriched genes for each tip population). To generate the tree structure, visitation frequencies were derived based on biased random walks performed from random tip cells towards the root.

URD functions were mostly performed with default parameters with the following exceptions: for the diffusion map calculation, the number of nearest neighbours (knn) was optimised to 200 and the kernel width to use (sigma.use) was set to 8. Regarding pseudotime calculations, a total of 150 simulations (floods) were performed and averaged. To determine tips for the tree structure, the optimal clustering of the E16.5 scRNA-seq data was found to be Louvain-Jaccard clustering with number of nearest neighbours (num.nn) set to 25. Clusters containing cells expressing progenitor markers were omitted from tip selection. To determine cellular trajectories in pseudotime, 10,000 biased random walks (n) were simulated. To build the tree, divergence method was set to “ks”; the remaining parameters were changed as follows: min.cells.per.segment = 10, p.thresh = 0.025, and minimum.visits = 1. To generate a force-directed layout, the method was set to “fr”, and the following arguments were modified: num.nn = 120, and min.final.neighbours = 4. Force-directed layout rendition of the URD tree then was optimised for visualisation in 2D using the “treeForceStretchCoords”, “treeForceRotateCoords”, and “treeForceTranslateCoords” functions from the URD package.

### Single-cell ATAC-seq

For single-cell ATAC-seq capture, ∼100,000 FACS-purified tdTomato^+^ cells from *Hic1*^*CreERT2*^; *R26*^*tdTomato*^ were collected from E12.5, E14.5, and E16.5 forelimbs as per scRNA-seq sorting conditions described above. Nuclei were isolated by lysing the cells according to the described protocol (10X Genomics, https://support.10xgenomics.com/single-cell-atac) for 5 min. Nuclei were quantified using a Countess II FL automated cell counter (ThermoFisher) and ∼10,000 nuclei were targeted for transposition and capture using a 10x Chromium controller. ScATAC-seq libraries were prepared according to the Chromium Single-Cell ATAC Reagent Kits User Guide (10x Genomics; CG000168 Rev B). Single-cell libraries were sequenced on a Nextseq500 (Illumina). The cellranger-atac single-cell ATAC pipeline version 1.1.0 was used to generate fastq files from the sequencer output bcl files and further perform read filtering and alignment, detection of accessible chromatin peaks, dimensionality reduction, cell clustering and differential accessibility analyses. Quality metrics for the scATAC-seq such as insert size distribution, enrichment around the transcriptional start site and a tSNE heatmap of fragments per cell (Supplementary Data 1) were obtained from the cellranger-atac output. Track plots were generated using Loupe Cell Browser (10x genomics, v. 4.1.0). The 10x genomics output was further processed using the standard Signac workflow to compute quality control metrics, normalisation, latent semantic indexing and subsequent UMAP calculation^47^. Cells were then clustered using the Leiden algorithm and integrated with scRNA-seq data using cross-modality integration and label transfer. For pseudotime and differential accessibility analysis, the 10x filtered peak barcode matrix output was used to generate a cell dataset-class object using monocle3. To generate pseudotime trajectories of chondrogenic, tenogenic and DF commitment, separate subset objects were created by using MP combined with chondrocyte, tenocyte or DF clusters, respectively. The cicero package was used to construct trajectories with accessibility data, perform differential accessibility analysis, and plot accessibility changes in pseudotime.

### Ex vivo forelimb cultures

Ex vivo culture of embryonic forelimbs was carried out as previously described^69^. Limb culture incubation chambers were prepared by placing a stainless-steel mesh into the inner well of a 60 mm organ culture dish. The stainless-steel mesh was previously punctured to allow for the placement of a PET membrane on its surface. The outer trough of the dish was filled with distilled water, and forelimb culture media (10% FBS in BGJb medium containing 100 mM penicillin/streptomycin and 100 mM l-glutamine) was added to the inner well. Embryonic limb buds were collected at E11.5 by dissection at the limb-trunk interface and placed on the PET membrane surface within a limb culture incubation chamber. Limbs were cultured in a well-humidified incubator (37 °C, 5% CO_2_) for 1–3 days. When live imaging was necessary, limbs were similarly cultured in a glass-bottom 35 mm dish atop a PET membrane and stainless-steel mesh.

### Micromass cultures

Preparation of primary limb mesenchyme cultures was done as previously described^70^. Briefly, E11.5 limb buds were dissected and enzymatically dissociated using a cocktail containing 10% FBS and Dispase I (10 units/mL) in Puck’s saline A. Limb buds were incubated in enzyme cocktail for 80 min at 37 °C with slight shaking (~70 rpm) and gently vortexed every 20 min. Following digestion, cells were washed in micromass media (10% FBS in 40% DMEM/60% F12 nutrient mixture with 100 mM penicillin/streptomycin and 100 mM l-glutamine), pelleted at 1000 x *g* for 5 min, filtered through a 40 µm cell strainer, and quantified using a haemocytometer. Micromass cultures were achieved by spotting 10 µL of cell solution at a density of 2 × 10^7^ cells/mL into the center of a well within a 24-well plate (Nunc or Grenier plates are optimal to allow the droplet to rest with minimal evaporation). Cultures were maintained in a humidified incubator (5% CO_2_) for an hour before adding 1 mL of micromass media to each well. Media was changed every other day thereafter.

### Quantification and statistical analysis

All data are represented as mean ± SD, the sample number and statistical analyses are indicated in the figure legends. Sample size determination was based on anticipated variability and effect size that was observed in the investigator’s lab for similar experiments. For pairwise comparisons, unpaired two-tailed *t*-tests were used to calculate *p*-values, *p* < 0.05. For comparison of >2 means, one-way ANOVA with Boneferroni’s correction was employed, *p* < 0.05. Both statistical methods were carried out using Prism (GraphPad Software v. 8).

### Reporting summary

Further information on research design is available in the Nature Research Reporting Summary linked to this article.

## Supplementary information

Supplementary Information

Description of Additional Supplementary Files

Supplementary Data 1

Supplementary Data 2

Supplementary Movie 1

Reporting Summary

## Data Availability

The scRNA-seq and scATAC-seq data generated in this study have been deposited in GEO under accession code GSE156953. Source data are provided within this paper.

## Source data

Source Data

## Code availability

All software used in this study is either freely or commercially available.
