# Supplementary material for: Cellular taxonomy of Hic1+ mesenchymal progenitor derivatives in the limb: from embryo to adult
Source: Nat Commun. 2022 Aug 25;13:4989. doi: 10.1038/s41467-022-32695-1 (PMC9411605; doi:10.1038/s41467-022-32695-1)
Supplement: Supplementary file 1 — Supplementary Information [file 41467_2022_32695_MOESM1_ESM.pdf]

Cellular taxonomy of Hic1^{+} mesenchymal progenitor derivatives in the limb; from embryo to adult

Supplementary Information

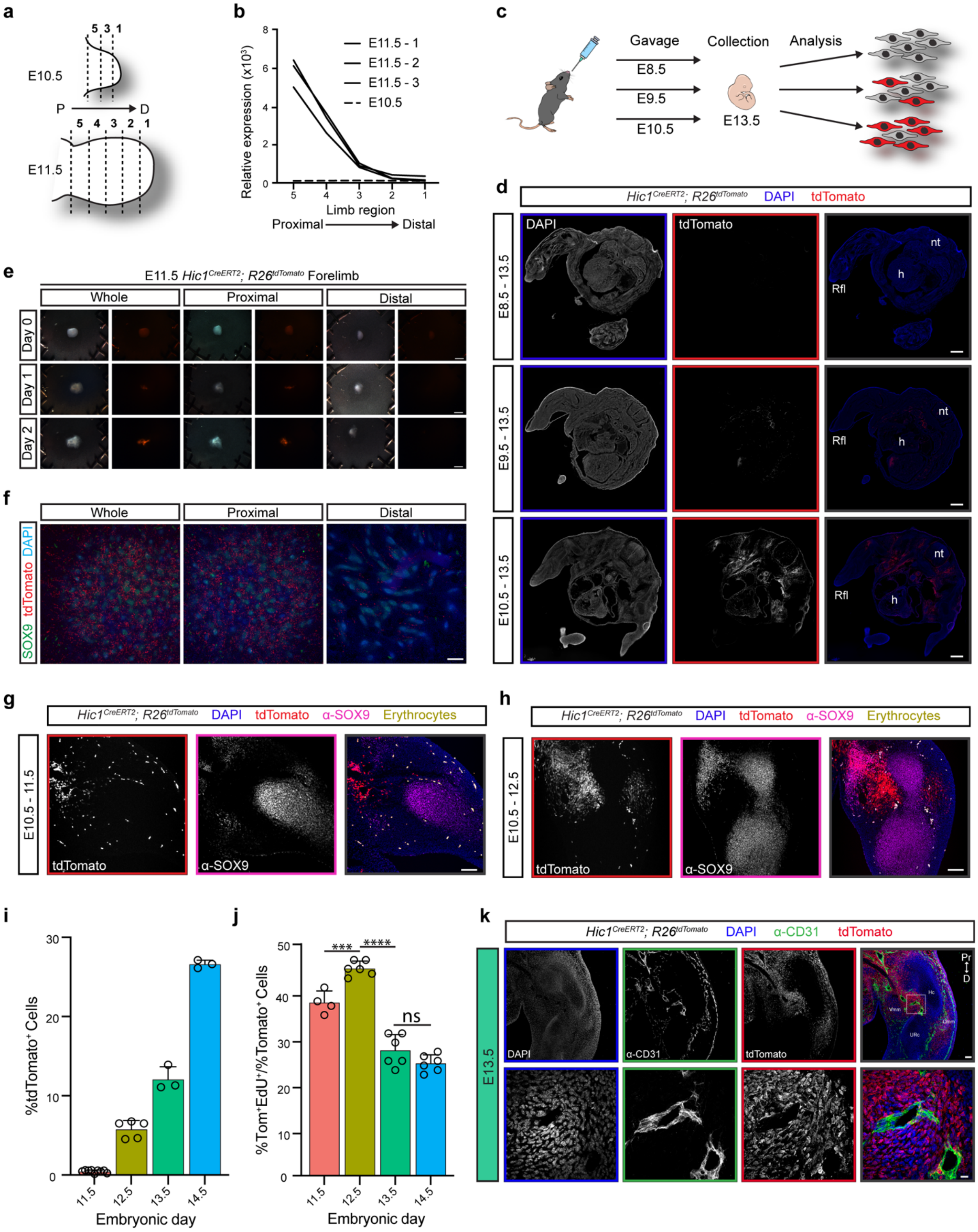
a
E10.5
E11.5
P
D
b
Relative expression (x10³)
Limb region
Proximal
Distal
E11.5 - 1
E11.5 - 2
E11.5 - 3
E10.5
c
Gavage
Collection
Analysis
E8.5
E9.5
E10.5
E13.5
d
Hic1CreERT2; R26tdTomato DAPI tdTomato
DAPI
tdTomato
E8.5 - 13.5
E9.5 - 13.5
E10.5 - 13.5
nt
h
Rfl
e
E11.5 Hic1CreERT2; R26tdTomato Forelimb
Whole
Proximal
Distal
Day 0
Day 1
Day 2
f
SOX9 tdTomato DAPI
g
Hic1CreERT2; R26tdTomato DAPI tdTomato α-SOX9 Erythrocytes
E10.5 - 11.5
α-SOX9
h
E10.5 - 12.5
i
%tdTomato+ Cells
Embryonic day
j
%Tom+EdU+/%Tomato+ Cells
ns
k
Hic1CreERT2; R26tdTomato DAPI α-CD31 tdTomato
E13.5
α-CD31
Pr
D

Supplementary Figure 1. Early expression dynamics of Hic1^{+} cells and progeny in the developing embryo. **(a-b)** Quantitative PCR analysis of *Hic1* transcripts, corresponding limb bud regions are shown in the left panel. Replicates indicated by 1-3 are shown. **(c)** Schematic representation of TAM temporal pulse optimization. **(d)** Fluorescence microscopy images of E13.5 *Hic1^{CreERT2}*; *R26^{tdTomato}* embryos treated at the indicated time points with a single TAM pulse. n = 3 embryos from independent litters. Scale bars, 500µm. h, heart; nt, neural tube; Rfl, right forelimb. **(e)** Representative wholemount images of *Hic1^{CreERT2}*; *R26^{tdTomato}* forelimbs cultured whole or proximally/distally-truncated and imaged at 0, 24, and 48 hours post collection. Scale bars, 1 mm. **(f)** Representative confocal maximum intensity projections of *Hic1^{CreERT2}*; *R26^{tdTomato}* forelimb micromass cultures; limbs were harvested at E11.5. n = 3 independent litters for whole limbs and 3 for proximal and distal portions. Scale bar, 100 µm. **(g)** Representative image of a *Hic1^{CreERT2}*; *R26^{tdTomato}* forelimb at E11.5, stained with α-SOX9 for identification of chondrogenic anlagen. n = 3 embryos from independent litters. Scale bar, 100 µm. **(h)** Representative image of a *Hic1^{CreERT2}*; *R26^{tdTomato}* forelimb at E12.5, stained with α-SOX9 for identification of chondrogenic anlagen. n = 3 embryos from independent litters. Scale bar, 100 µm. **(i)** FCM analysis of enzymatically dissociated embryo forelimbs from *Hic1^{CreERT2}*; *R26^{tdTomato}* embryos at specified developmental timepoints. n = 3 independent litters per timepoint. Data represent mean ± SD. **(j)** FCM analysis of EdU incorporation assay on *Hic1^{CreERT2}*; *R26^{tdTomato}* forelimbs. n = 4-6 independent litters per timepoint. Data represent mean ± SD, one-way ANOVA followed by Tukey's multiple comparison test, nsP=0.2247, ***P=0.00095, ****P<0.0001. **(k)** Representative images of *Hic1^{CreERT2}*; *R26^{tdTomato}* forelimbs at E13.5, counterstained with CD31 and DAPI. n = 3 embryos from independent litters. Scale bars, 100µm (left half), 20 mm (right half). D, distal; Dmm, dorsal muscle mass; Hc, pre-cartilage primordium of humerus; Pr, proximal; URc, radio-ulnar pre-cartilage primordium; Vmm, ventral muscle mass. Source data are provided as a Source Data file.

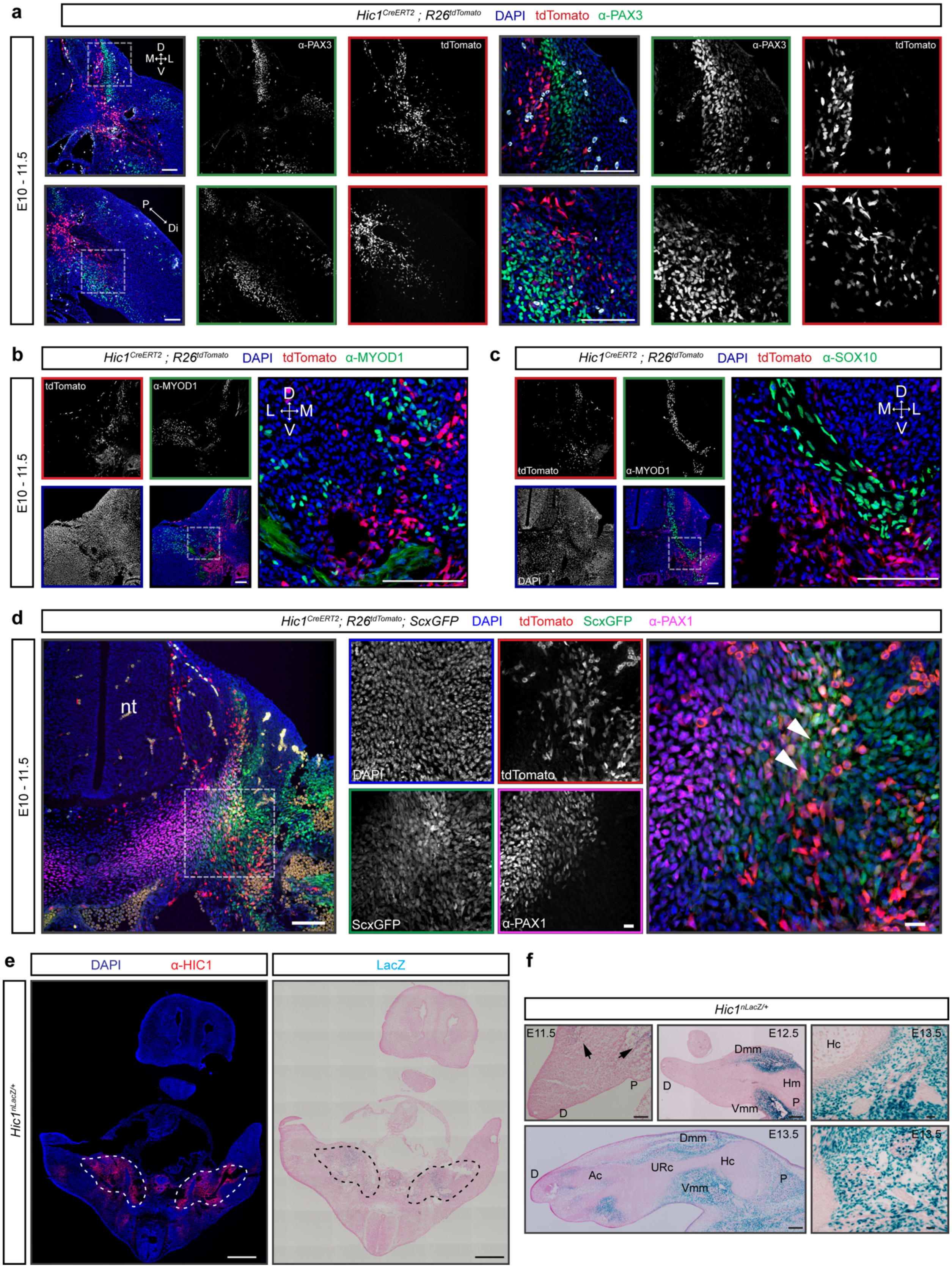

a
Hic1CreERT2 ; R26tdTomato DAPI tdTomato α-PAX3
E10 - 11.5
D M L V
α-PAX3
tdTomato
P Di
b
Hic1CreERT2 ; R26tdTomato DAPI tdTomato α-MYOD1
tdTomato
α-MYOD1
D L M V
c
Hic1CreERT2 ; R26tdTomato DAPI tdTomato α-SOX10
tdTomato
α-MYOD1
DAPI
d
Hic1CreERT2; R26tdTomato; ScxGFP DAPI tdTomato ScxGFP α-PAX1
nt
DAPI
tdTomato
ScxGFP
α-PAX1
e
DAPI α-HIC1
LacZ
Hic1nLacZ/+
f
Hic1nLacZ/+
E11.5
P
D
E12.5
Dmm
Hm
Vmm
E13.5
Hc
Ac
URc

Supplementary Figure 2. Characterization of *Hic1*/HIC1^{+} MP spatial distribution in the developing embryo. **(a-c)** Immunofluorescence staining with α-PAX3 (a), α-MYOD1 (b), and α-SOX10 on E11.5 *Hic1*^{CreERT2}; *R26*^{tdTomato} embryos at the forelimb level. Dotted boxes indicate inset regions on adjoining panels. Scale bars, 100 µm. D, dorsal; Di, distal; L, lateral; M, medial; P, proximal; V, ventral. **(d)** Immunofluorescence staining with α-PAX1 on E11.5 *Hic1*^{CreERT2}; *R26*^{tdTomato}; *Scx-GFP* embryos at the forelimb level. Dotted boxes indicate inset region on adjoining panels. Scale bars, 100 µm, 20 µm inset. Nt, neural tube. **(e)** Representative images of *Hic1*$^{nLacZ/+}$ E11.5 embryo sections, left panel is stained with α-HIC1, right panel contains subjacent section stained with X-gal and NFR, example overlapping region highlighted. Scale bar, 500µm. **(f)** Images of transverse sections from *Hic1*$^{nLacZ/+}$ embryonic forelimbs at the indicated timepoints stained for nuclear LacZ expression. Sections were counterstained with NFR. Black arrowheads highlight two groups of LacZ^{+} cells within the proximal end of the forelimb. Scale bars, 100 µm. n = 3 embryos from independent litters for panels a-f. Hm, humeral mesenchymal condensation; RUm, radio-ulnar mesenchymal condensation; Hc, pre-cartilage primordium of humerus; URc, radio-ulnar pre-cartilage primordium; Dmm, dorsal muscle mass; Vmm, ventral muscle mass.

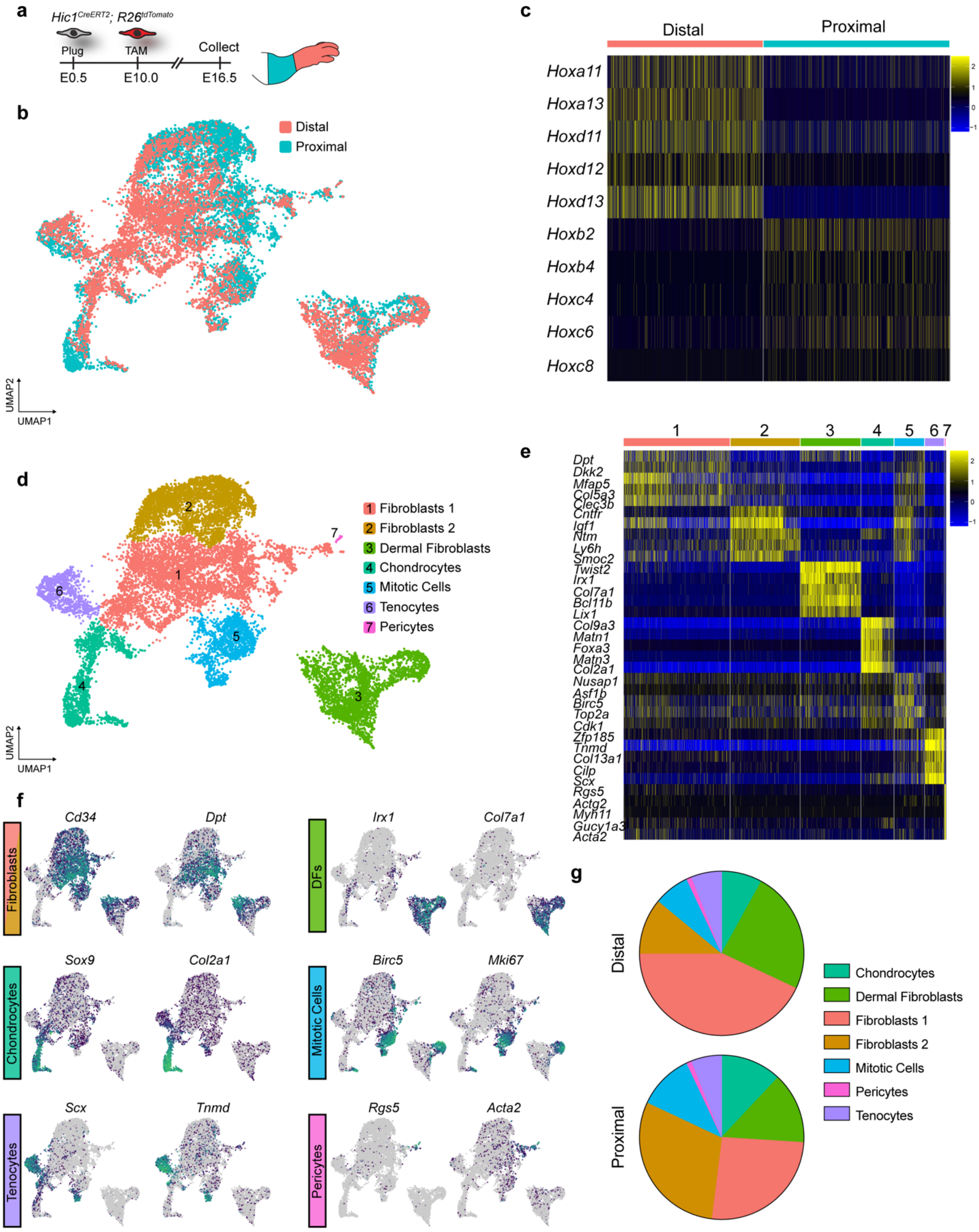

a
Hic1CreERT2; R26tdTomato
Plug
TAM
Collect
E0.5
E10.0
E16.5
b
Distal
Proximal
UMAP2
UMAP1
c
Distal
Proximal
Hoxa11
Hoxa13
Hoxd11
Hoxd12
Hoxd13
Hoxb2
Hoxb4
Hoxc4
Hoxc6
Hoxc8
d
1 Fibroblasts 1
2 Fibroblasts 2
3 Dermal Fibroblasts
4 Chondrocytes
5 Mitotic Cells
6 Tenocytes
7 Pericytes
UMAP2
UMAP1
e
1 2 3 4 5 6 7
Dpt
Dkk2
Mfap5
Col5a3
Clec3b
Cntfr
Igf1
Ntm
Ly6h
Smoc2
Twist2
Irx1
Col7a1
Bcl11b
Lix1
Col9a3
Matn1
Foxa3
Matn3
Col2a1
Nusap1
Asf1b
Birc5
Top2a
Cdk1
Zfp185
Tnmd
Col13a1
Cilp
Scx
Rgs5
Actg2
Myh11
Gucy1a3
Acta2
f
Fibroblasts
Cd34
Dpt
DFs
Irx1
Col7a1
Chondrocytes
Sox9
Col2a1
Mitotic Cells
Birc5
Mki67
Tenocytes
Scx
Tnmd
Pericytes
Rgs5
Acta2
g
Distal
Proximal
Chondrocytes
Dermal Fibroblasts
Fibroblasts 1
Fibroblasts 2
Mitotic Cells
Pericytes
Tenocytes

Supplementary Figure 3. ScRNA-seq analysis of proximal and distal cell fates in E16.5 *Hic1^{CreERT2}; R26^{tdTomato}* forelimbs. (a) Schematic representation of the experimental design. **(b)** UMAP of aggregated proximal and distal data. **(c)** Gene expression heatmap of relevant Hox-genes for proximal-distal axis formation. **(d)** UMAP of aggregated data coloured by cluster. **(e)** Heatmap of scRNA-seq data showing enriched genes within each cluster. **(f)** UMAPs of representative genes for each identified cell population. **(g)** Pie charts representing the distribution of fates for cells isolated from the distal and proximal end of an E16.5 forelimb. Source data are provided as a Source Data file.

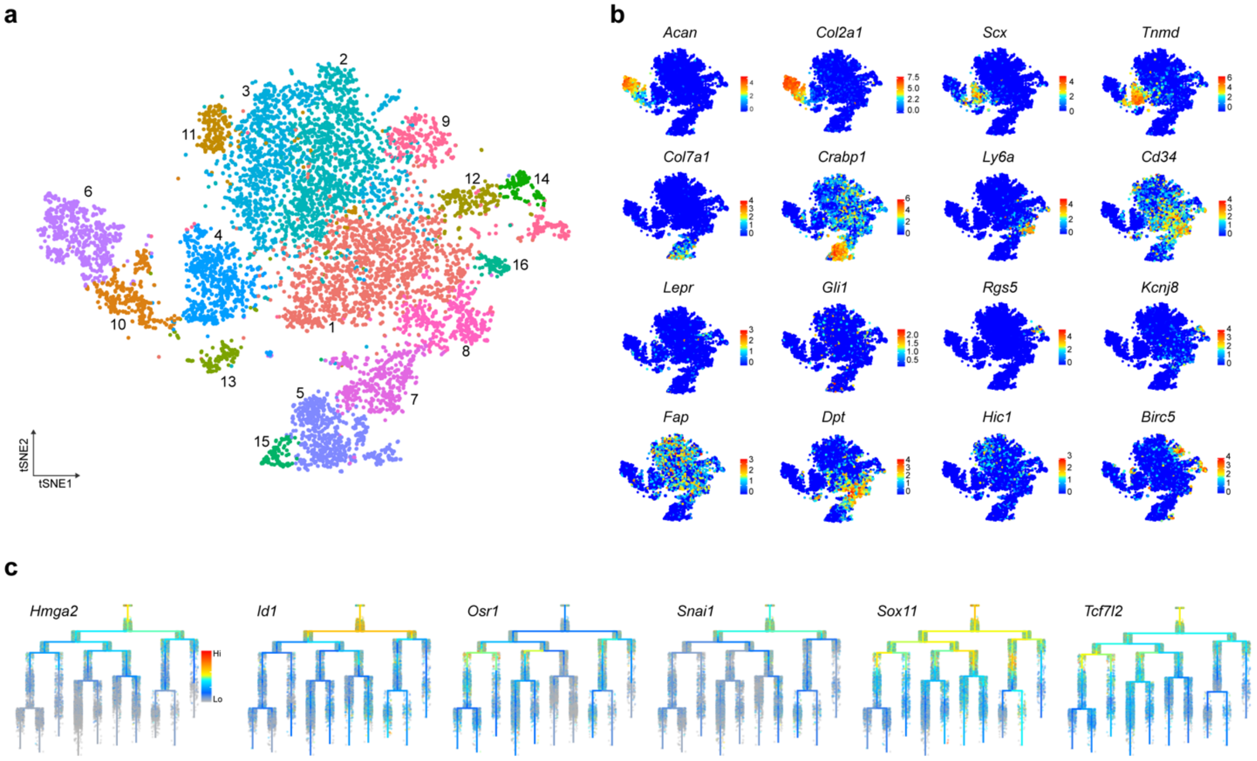

Supplementary Figure 4. ScRNA-seq analyses reveal multiple lineage potentials. (a) Cluster map of E16.5 *Hic1*^{CreERT2}; *R26*^{tdTomato} sorted tdTomato^{+} cells projected in URD-generated t-SNE space. **(b)** Representative genes used to assign URD cluster identities and further determine the tips of the developmental trajectories. **(c)** Dendrograms illustrating gene expression intensity of progenitor-associated genes projected on the mesenchymal lineage hierarchy.

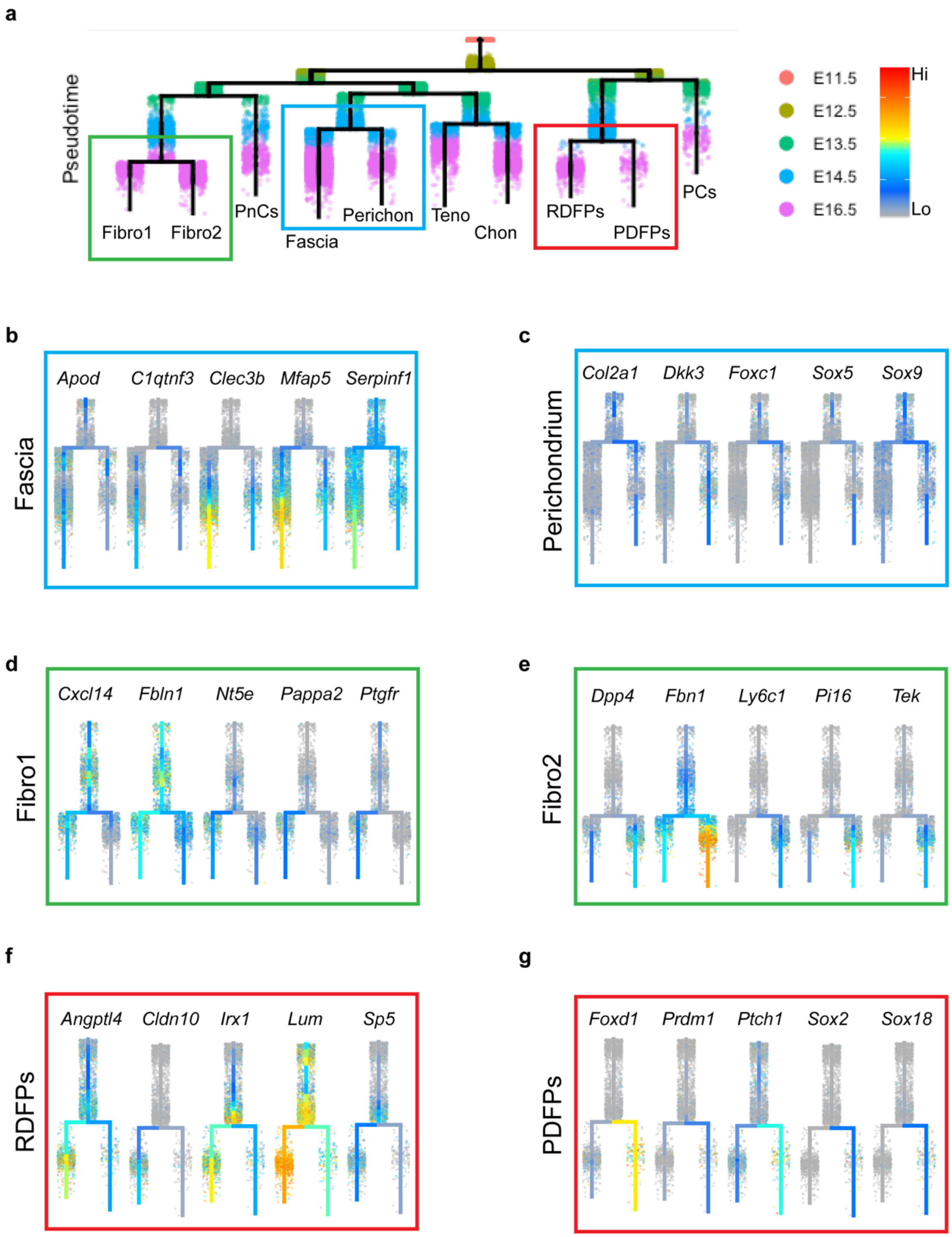

Supplementary Figure 5. Characterization of embryonic *Hic1*⁺ MP-derived fibroblast populations. (a) URD-inferred developmental trajectories of *Hic1* lineage-labelled forelimb MPs, fibroblast populations are highlighted by coloured boxes. **(b-g)** Gene expression heatmaps projected on dendrogram branchpoints for each fibroblast subpopulation. Fibro1, fibroblast 1; Fibro2, fibroblast 2, RDFPs, reticular dermal fibroblast progenitors; PDFPs, papillary dermal fibroblast progenitors.

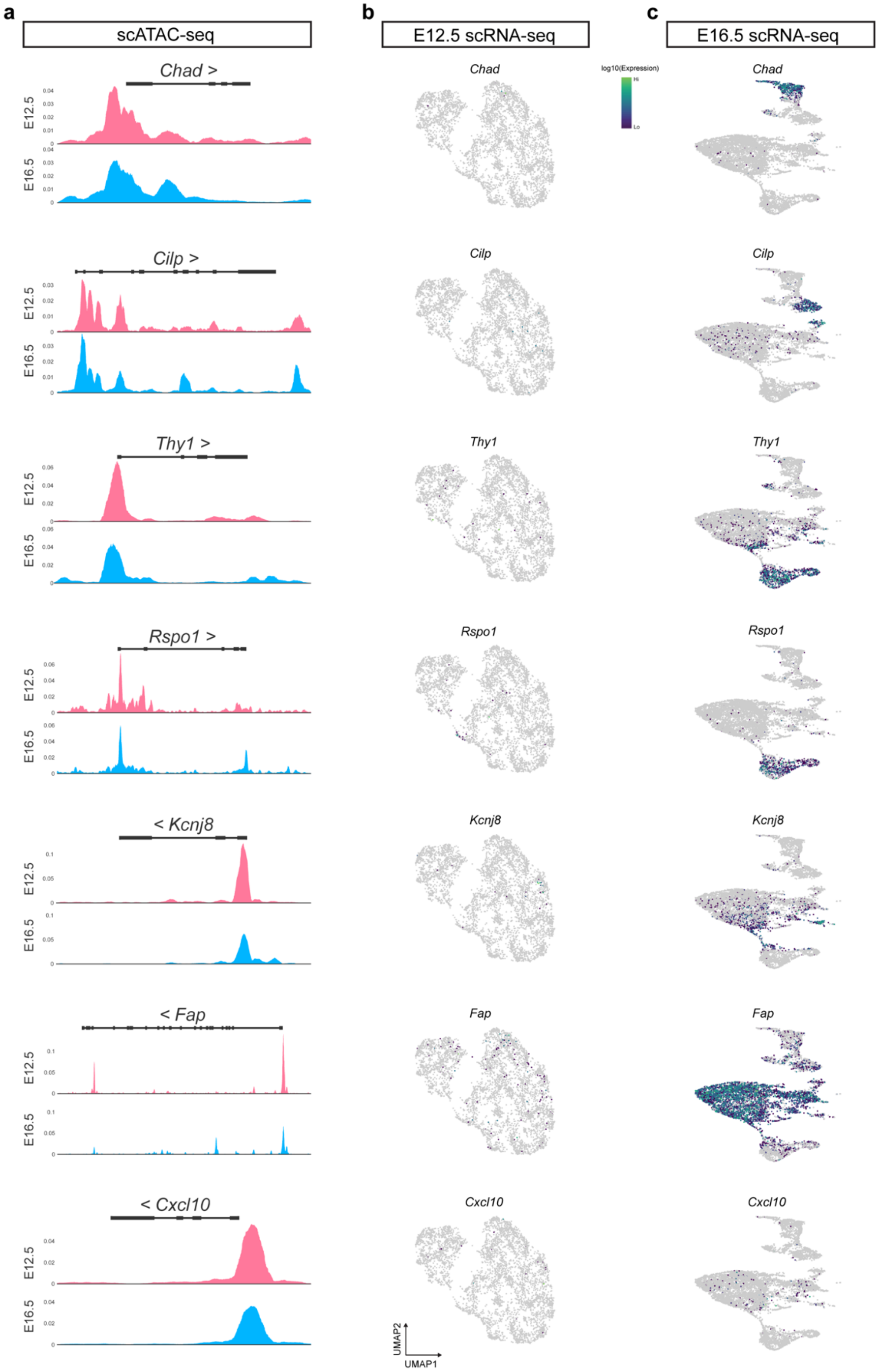

Supplementary Figure 6. Broad lineage potential is embedded in the epigenome of early MPs. **(a)** genome browser tracks displaying the promoter sum signals for diverse lineage-associated genes from scATAC-seq analyses of E12.5 and E16.5 samples. **(b-c)** UMAP plots of scRNA-seq expression data of genes in panel a. b, E12.5; c, E16.5.

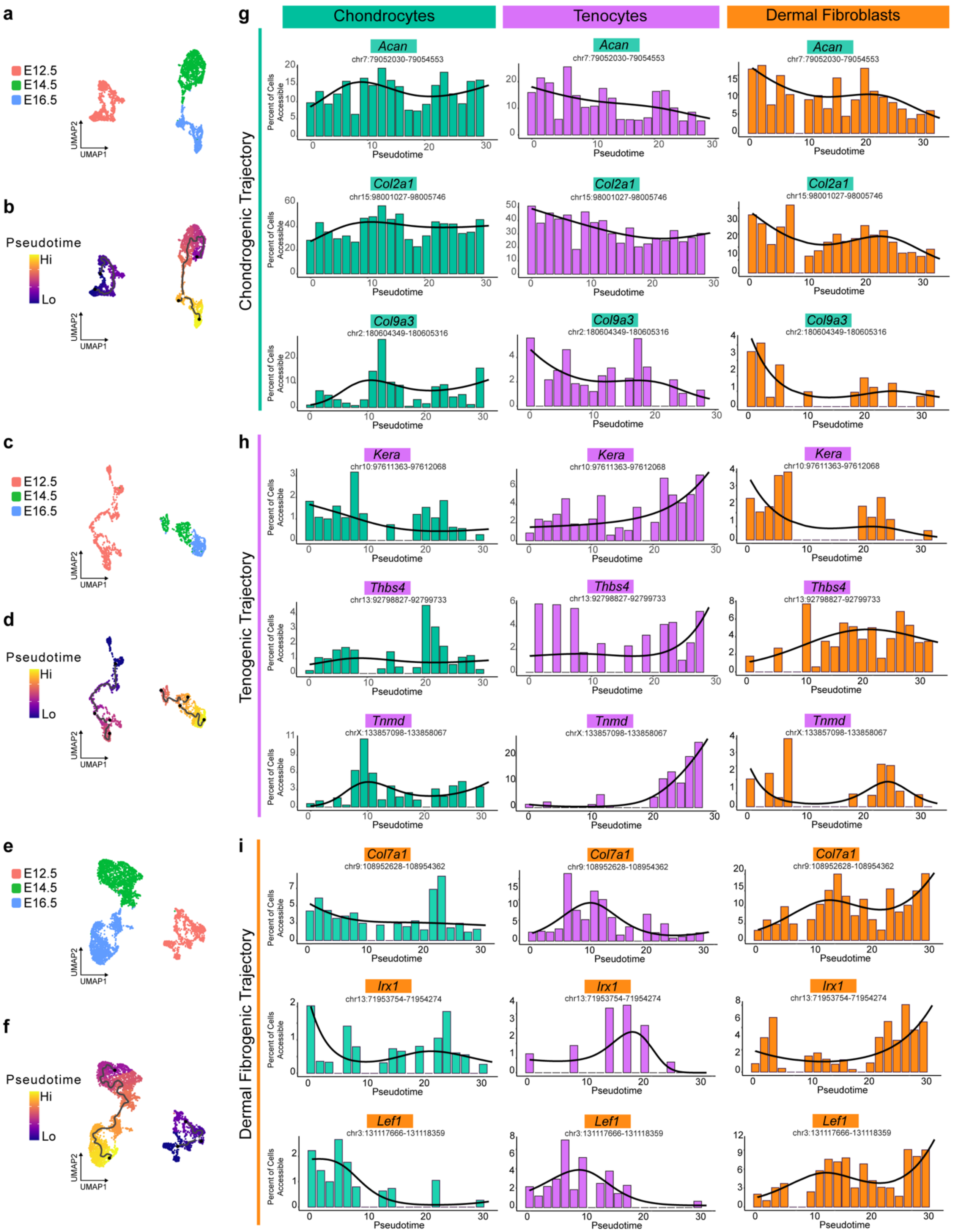
a
E12.5
E14.5
E16.5
UMAP2
UMAP1
b
Pseudotime
Hi
Lo
c
d
e
f
g
h
i
Chondrocytes
Tenocytes
Dermal Fibroblasts
Chondrogenic Trajectory
Tenogenic Trajectory
Dermal Fibrogenic Trajectory
Acan
chr7:79052030-79054553
Col2a1
chr15:98001027-98005746
Col9a3
chr2:180604349-180605316
Kera
chr10:97611363-97612068
Thbs4
chr13:92798827-92799733
Tnmd
chrX:133857098-133858067
Col7a1
chr9:108952628-108954362
Irx1
chr13:71953754-71954274
Lef1
chr3:131117666-131118359
Percent of Cells Accessible
Pseudotime

Supplemental Figure 7. Pseudotime trajectory reconstruction of scATAC-seq embryonic forelimb datasets reveals a progressive restriction of OCRs at fate-specific promoters. (a-b) UMAP plot of scATAC-seq chondrocyte and MP cluster subset coloured by embryonic time point and pseudotime values. **(c-d)** UMAP plot of scATAC-seq tenocyte and MP cluster subsets coloured by embryonic time point and pseudotime values. **(e-f)** UMAP plot of scATAC-seq dermal fibroblast and MP cluster subset coloured by embryonic time point and pseudotime values. **(g-i)** Chromatin differential accessibility graphs for fate-specific gene markers in the chondrocyte (g), tenocyte (h), and dermal fibroblast (i) trajectories.

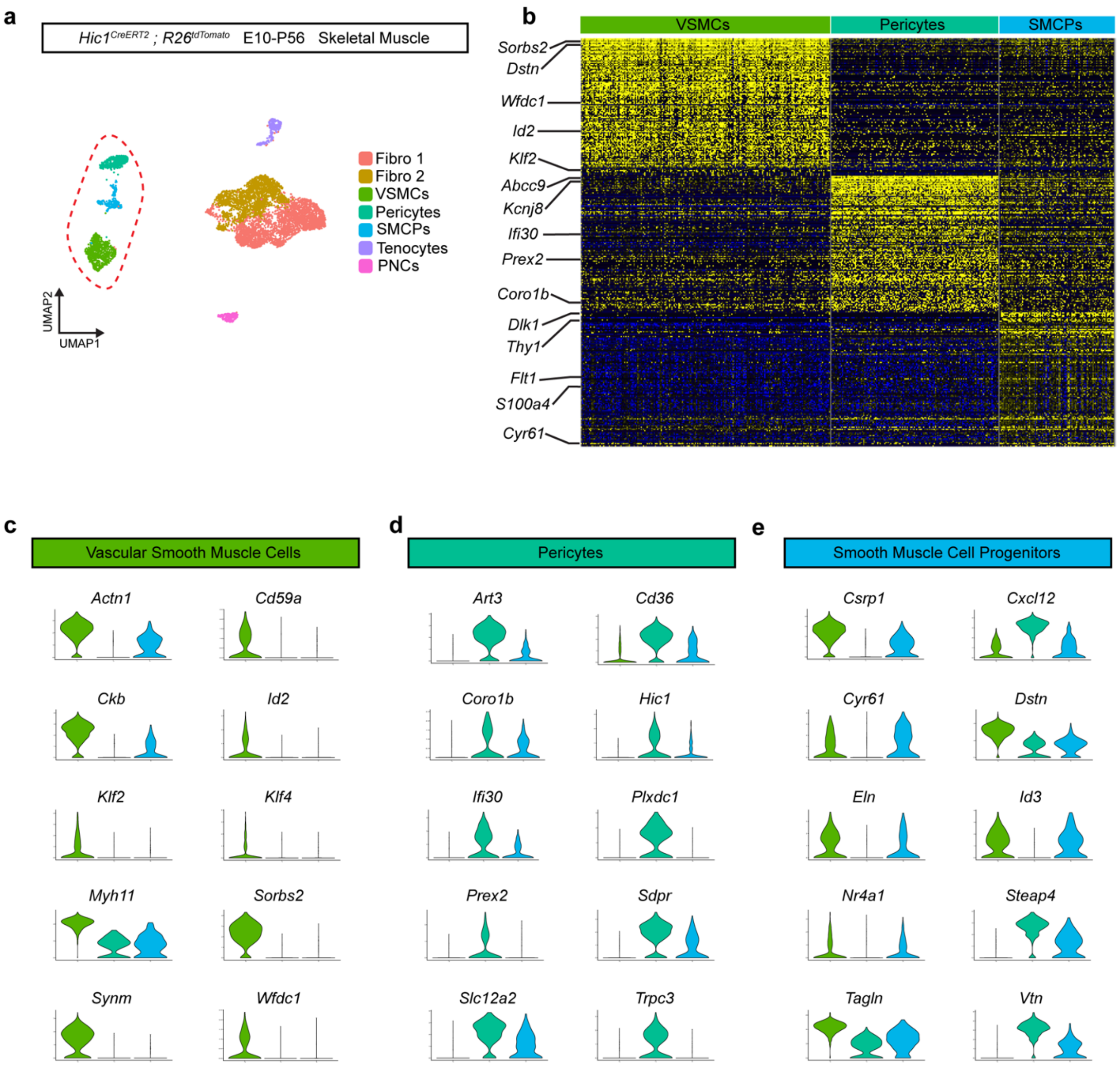

Supplementary Figure 8. Characterization of mural cell fates. (a) UMAP coloured by cluster of scRNA-seq data from sorted tdTomato$^+$ cells isolated from the TA muscle of lineage-traced *Hic1*^{CreERT2}*; R26*^{tdTomato} mice. Dotted line encircles endothelium-associated clusters (VSMCs, pericytes, and SMCPs). **(b)** Heatmap of differentially expressed genes between VSMCs, pericytes, and SMCPs clusters. **(c-d)** Violin plots for expression of representative genes from each cluster indicated in (a).

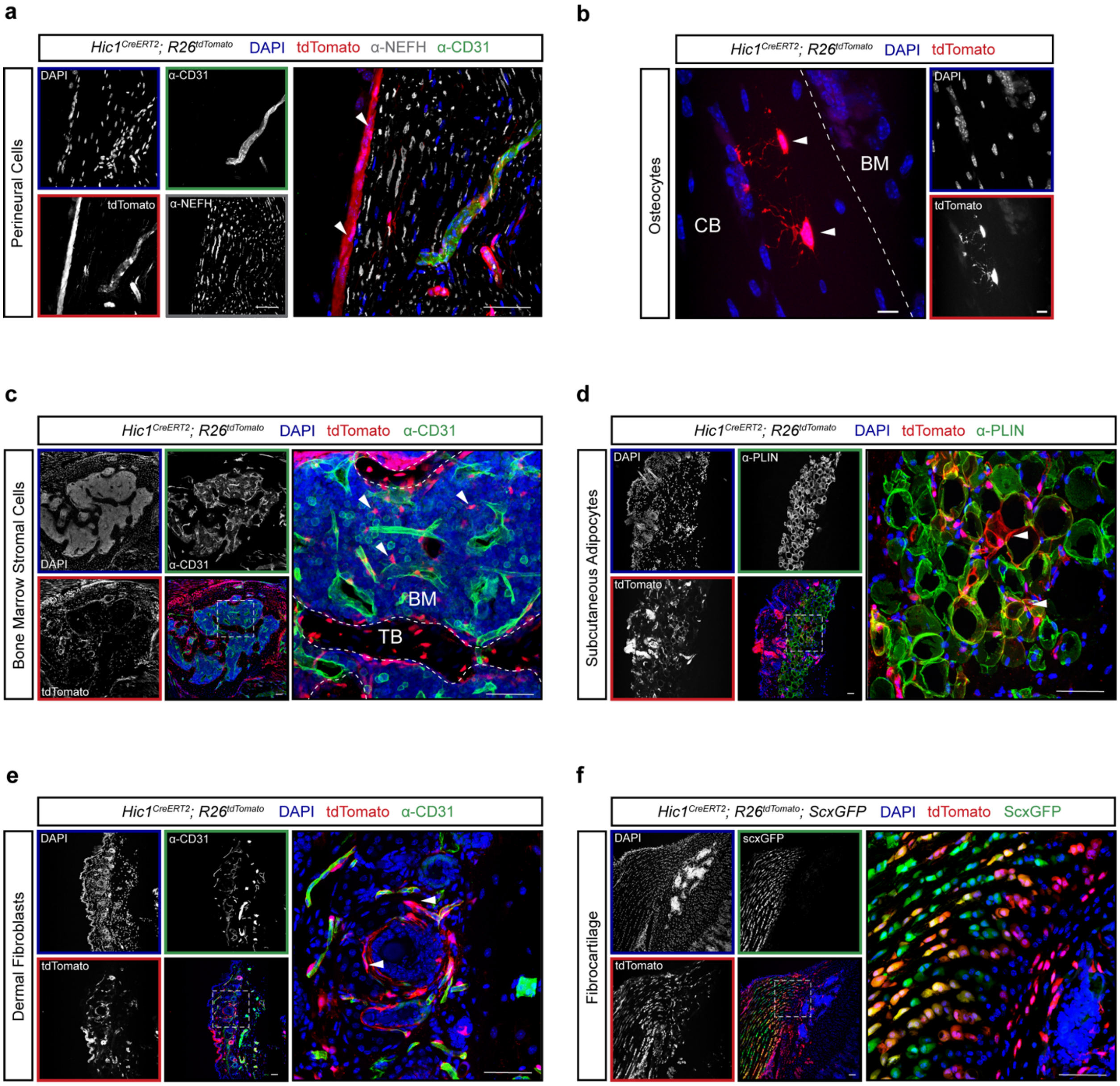

Supplementary Figure 9. Embryonic Hic1$^+$ MPs contribute to diverse cell types that persist into adulthood. (a-f) Representative immunofluorescence images of E10-P56 limb tissues demonstrating contribution of Hic1 MPs to the following lineages: Perineural cells (a); osteocytes (b); bone marrow stromal cells (c), subcutaneous adipocytes (d), dermal fibroblasts (e); and fibrocartilage (f). White arrowheads indicate cells of interest. White boxes in (c, e, f) highlight regions of interest that are magnified in adjoining panels. Scale bars, 20µm. n = 3 mice for panels a-f. BM, bone marrow; CB, cortical bone; TB, trabecular bone.

a Gating strategy for tdTomato⁺ cell quantification experiments

b Gating strategy for EdU-incorporation experiments

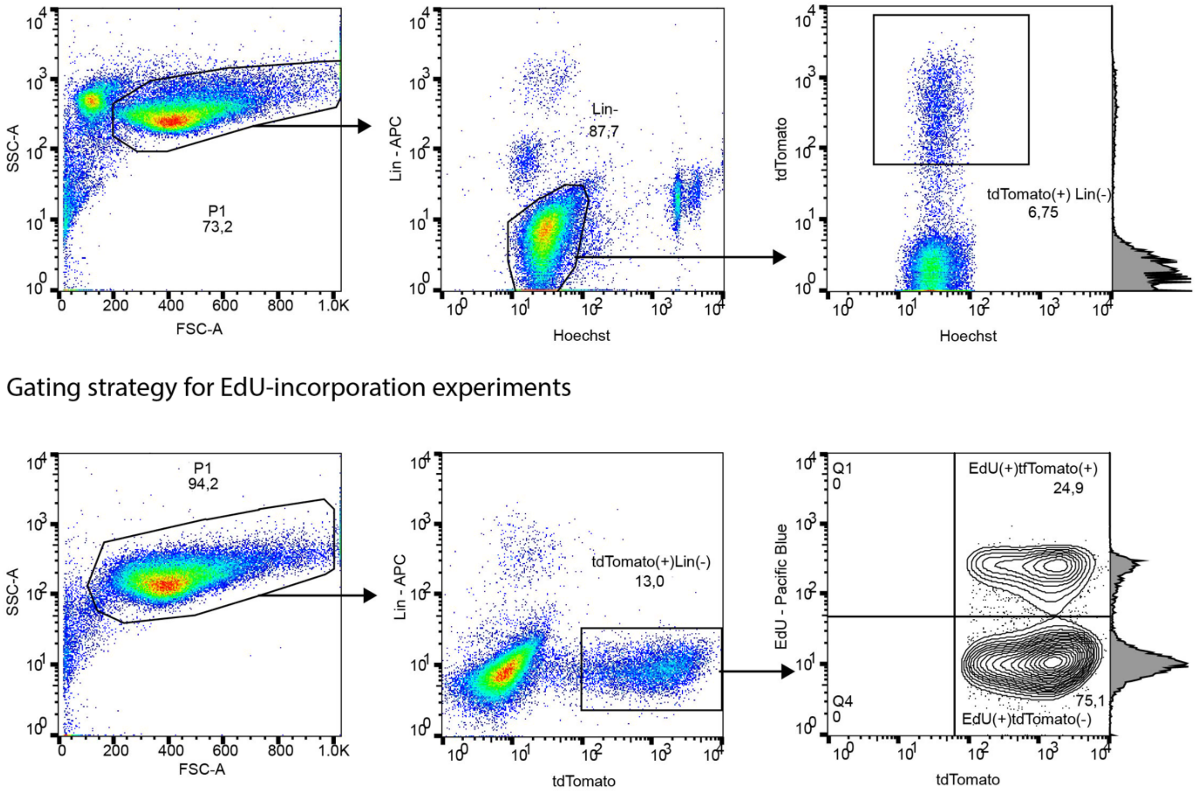

Supplementary Figure 10. Examples of gating strategies used. (a) Example of FCM plots used to quantify $tdTomato^{+}$ cells. **(b)** Example of FCM plots used to quantify $tdTomato^{+}/EdU^{+}$ cells.
