# Supplementary material for: Cellular taxonomy of Hic1+ mesenchymal progenitor derivatives in the limb: from embryo to adult
Source: Nat Commun. 2022 Aug 25;13:4989. doi: 10.1038/s41467-022-32695-1 (PMC9411605; doi:10.1038/s41467-022-32695-1)
Supplement: Supplementary file 2 — Description of Additional Supplementary Files [file 41467_2022_32695_MOESM2_ESM.pdf]

Description of Additional Supplementary Files

Supplementary Data 1. Quality metrics for scRNA- and scATAC-seq data generated in this work.

Supplementary Data 2. Top 500 enriched genes per tip or the URD-generated cell lineage tree.

Supplementary Movie 1. Monochromatic rotating volume of E11.75 $Hic1^{CreERT2}$; $R26^{tdTomato}$ embryo.
