# Supplementary material for: Cellular taxonomy of Hic1+ mesenchymal progenitor derivatives in the limb: from embryo to adult
Source: Nat Commun. 2022 Aug 25;13:4989. doi: 10.1038/s41467-022-32695-1 (PMC9411605; doi:10.1038/s41467-022-32695-1)
Supplement: Supplementary file 6 — Reporting Summary [file 41467_2022_32695_MOESM6_ESM.pdf]

nature research

Corresponding author(s): Underhill, T.M.

Last updated by author(s): 7/15/2022

Reporting Summary

Nature Research wishes to improve the reproducibility of the work that we publish. This form provides structure for consistency and transparency in reporting. For further information on Nature Research policies, see our Editorial Policies and the Editorial Policy Checklist.

Statistics

For all statistical analyses, confirm that the following items are present in the figure legend, table legend, main text, or Methods section.

n/a	Confirmed	
☐	☒	The exact sample size (*n*) for each experimental group/condition, given as a discrete number and unit of measurement
☒	☐	A statement on whether measurements were taken from distinct samples or whether the same sample was measured repeatedly
☐	☒	The statistical test(s) used AND whether they are one- or two-sided<br>*Only common tests should be described solely by name; describe more complex techniques in the Methods section.*
☒	☐	A description of all covariates tested
☐	☒	A description of any assumptions or corrections, such as tests of normality and adjustment for multiple comparisons
☐	☒	A full description of the statistical parameters including central tendency (e.g. means) or other basic estimates (e.g. regression coefficient) AND variation (e.g. standard deviation) or associated estimates of uncertainty (e.g. confidence intervals)
☐	☒	For null hypothesis testing, the test statistic (e.g. F, t, r) with confidence intervals, effect sizes, degrees of freedom and P value noted<br>*Give P values as exact values whenever suitable.*
☒	☐	For Bayesian analysis, information on the choice of priors and Markov chain Monte Carlo settings
☒	☐	For hierarchical and complex designs, identification of the appropriate level for tests and full reporting of outcomes
☒	☐	Estimates of effect sizes (e.g. Cohen's d, Pearson's r), indicating how they were calculated

Our web collection on statistics for biologists contains articles on many of the points above.

Software and code

Policy information about availability of computer code

Data collection	Flow cytometry data were collected using BD FACS Diva 6.1.3. Cells were sorted using BC FACS Sortware 1.0.0.650. Immunofluorescence data were collected using CellSens Dimension 1.18 and NIS-Elements 4.60.00, brightfield wholemount images were collected using Q-capture Pro 7.0.30 software.
Data analysis	Cellranger 4.0.0 was used to align, enumerate, and analyze single cell data. Seurat 3.2.1, monocle3 0.2.0, Signac 1.1.0, and Cicero 1.3.4.11 R packages were used for single cell RNA and ATAC-seq analysis and visualization. URD 1.1.0.9005 R package was used for reconstruction of single cell transcriptional trajectories. ImageJ V 2.0.0 was used for image processing. FlowJo 10.6.1 was used to analyze and visualize all cytometer and FACS data. GraphPad Prism 8.4.3 was used for all statistical analyses. Loupe Browser 4.1.0 was used to analyse and generate scATAC-seq genome browser tracks. Seurat implements the Leiden algorithm (R package version 0.3.9) to identify cell clusters in scRNA-seq.

For manuscripts utilizing custom algorithms or software that are central to the research but not yet described in published literature, software must be made available to editors and reviewers. We strongly encourage code deposition in a community repository (e.g. GitHub). See the Nature Research guidelines for submitting code & software for further information.

Data

Policy information about availability of data

All manuscripts must include a data availability statement. This statement should provide the following information, where applicable:

- Accession codes, unique identifiers, or web links for publicly available datasets
- A list of figures that have associated raw data
- A description of any restrictions on data availability

Single cell RNA and ATAC-seq data generated in this manuscript are available at GEO repository accession number GSE156953. The skeletal muscle scRNA-seq dataset from adult lineage-traced Hic1CreERT2;R26tdTomato mice used in Figure 8 is publicly available at GEO repository with accession number GSE110038.

Field-specific reporting

Please select the one below that is the best fit for your research. If you are not sure, read the appropriate sections before making your selection.

☒ Life sciences ☐ Behavioural & social sciences ☐ Ecological, evolutionary & environmental sciences

For a reference copy of the document with all sections, see nature.com/documents/nr-reporting-summary-flat.pdf

Life sciences study design

All studies must disclose on these points even when the disclosure is negative.

Sample size	No sample size calculation was performed. Sample size was determined from similar experiments in the literature and for all experiments a minimum of three biological replicates were analyzed per time-point or condition with the exception of single cell RNA-seq analyses.
Data exclusions	High mitochondrial RNA and low UMI counts were used as standards to exclude low-quality cells from downstream analyses in the scRNA-Seq experiments. Furthermore, during the construction of pseudotime trajectories using URD, the scRNA-seq dataset was further refined to exclude proliferating and/or progenitor cells from the tips of the tree.
Replication	All findings were reproduced in multiple independent experiments with the exception of single cell capture. Data in this study is presented in most cases as an aggregate of all experiments. The number of biological replicates for each data panel is indicated in the respective figure legend. Immunofluorescence images and µCT reconstructions are displayed as representative examples.
Randomization	Timed pregnancies were arranged according to genotype and resulting litters were allocated randomly to experiments and processed in an arbitrary order.
Blinding	For analysis of cell numbers after NTX damage in skeletal muscle of adult vs embryonic lineage traced mice where comparative analyses were performed, researchers were blinded by giving samples an arbitrary code for cell counting and imaging, and samples were unblinded post-data collection.

Reporting for specific materials, systems and methods

We require information from authors about some types of materials, experimental systems and methods used in many studies. Here, indicate whether each material, system or method listed is relevant to your study. If you are not sure if a list item applies to your research, read the appropriate section before selecting a response.

Materials & experimental systems

n/a	Involved in the study	
☐	☒	Antibodies
☒	☐	Eukaryotic cell lines
☒	☐	Palaeontology and archaeology
☐	☒	Animals and other organisms
☒	☐	Human research participants
☒	☐	Clinical data
☒	☐	Dual use research of concern

Methods

n/a	Involved in the study	
☒	☐	ChIP-seq
☐	☒	Flow cytometry
☒	☐	MRI-based neuroimaging

Antibodies

Antibodies used

The following antibodies were used in this study:
Anti-Myosin heavy chain, sarcomere (MF20), clone: P3U-1, Cat. #MF 20, Lot: 7/6/17, DSHB, 1:50
Anti-MYOD1, clone: EPR6653-131, Cat. #ab133627, Lot: GR3375193-2 , Abcam, 1:100
Anti-PAX1, clone: 5A2, Cat. #ab252847, Lot: GR3345842-1, Abcam, 1:100
Anti-PAX3, clone: C2, Cat. #Pax3, DSHB, 1:50
Anti-SOX10, clone: EPR4067, Cat. #ab155279, Lot: GR113617-36, Abcam, 1:100
Anti-SOX9, clone: EPR14335-78, Cat. #ab185966, Lot: GR3241181-3, Abcam, 1:100
Anti-CD31, clone: MEC 13.3, Cat. #550274, Lot: 8079850, BD Pharmingen, 1:50
Anti-myosin (skeletal, fast), clone: MY-32, Cat. #M4276, Lot: 105M4841V, Sigma Aldrich, 1:1000
Anti-PAX7, clone: PAX-7, Cat. AB0000456, UBC Ablab, 1:200
Anti-Neurofilament heavy chain, Cat. #ab4680, Lot: GR3241438-11, Abcam, 1:5000
Anti-RFP, Cat. #ab62341, Lot: GR3184770-3, Abcam, 1:100
Anti-GFP, Cat. #ab13970, Lot. GR236651-21, Abcam, 1:100
Anti-COL22A1, Cat. #ab121846, Lot. GR223910-6, Abcam, 1:100
Anti-HIC1 C25, Provided by Underhill Lab, 1:4000
Anti-CD31-AlexaFluor647, clone: 390, Cat. #67-0032-01, Lot: AB0000998, UBC Ablab, 1:400

Anti-CD117-APC, clone: ACK2, Cat. #17-1172-82, Lot: 2183542, eBioscience, 1:500
Anti-CD11B-AlexaFluor647, clone: M1/70, Cat. #67-0055-01, Lot: AB0000897, UBC Ablab, 1:500
Anti-F4/80-AlexaFluor647, clone: BM8, Cat. #67-0035-05, Lot: AB0001029, UBC Ablab, 1:500
Anti-CD45-AlexaFluor647, clone: I3/2, Cat. #67-0047-01, Lot: AB0000935, UBC ABlab, 1:400
Anti-Ter119-AlexaFluor647, clone: TER119, Cat. #67-0031-01, Lot: AB00000305, UBC Ablab, 1:200
Alexa Fluor 488 goat anti-mouse IgG, Cat. #A11029, Lot: 1252783, Thermo Fisher Scientific, 1:500
Alexa Fluor 647 goat anti-mouse IgG, Cat. #A32728, Lot: VH311610, Thermo Fisher Scientific, 1:500
Alexa Fluor 647 goat anti-rat IgG, Cat. #A21247, Lot: 2089926, Thermo Fisher Scientific, 1:500
Alexa Fluor 594 goat anti-rabbit IgG, Cat. #A11012, Lot: 2018240, Thermo Fisher Scientific, 1:500
Alexa Fluor 488 goat anti-rabbit IgG, Cat. #A11034, Lot: 2018207, Thermo Fisher Scientific, 1:500
Alexa Fluor 647 goat anti-rabbit IgG, Cat. #A27040, Lot: RB232998A, Thermo Fisher Scientific, 1:500
Alexa Fluor 488 goat anti-rat IgG, Cat. #A11006, Lot: 2048174, Thermo Fisher Scientific, 1:500
Alexa Fluor 488 goat anti-chicken IgG, Cat. #A11039, Lot: 1937504, Thermo Fisher Scientific, 1:500

Validation

The HIC1-C25 antibody was validated by immunofluorescence staining of Hic1-null tissue samples and western blot of Hic1-null primary fibroblasts.

Anti-Myosin heavy chain, sarcomere (MF20) (Cat. #MF 20, DSHB). This monoclonal antibody has been tested to recognize all MHC isoforms of mammals. For more information, visit https://dshb.biology.uiowa.edu/MF-20.

Anti-MYOD1 (Cat. #ab133627, Abcam). This monoclonal antibody has been validated by testing with a human muscle rhabdomyosarcoma cell line. https://www.abcam.com/myod1-antibody-epr6653-131-ab133627.html

Anti-PAX1 Cat. #ab252847, Abcam. This recombinant monoclonal antibody has been validated by testing on mouse E14.5 embryonic tissue. https://www.abcam.com/pax1-antibody-5a2-ab252847.html

Anti-PAX3 (Cat. #Pax3, DSHB). This monoclonal antibody has been validated by detection of the developing placodes in quail embryos and is cross-reactive with mice. For more information see https://dshb.biology.uiowa.edu/Pax3.

Anti-SOX10 C(at. #ab155279 Abcam). This monoclonal antibody has been validated by the manufacturer through detection of SOX10 in C6 cells. https://www.abcam.com/sox10-antibody-epr4007-ab155279.html

Anti-PAX7, clone: PAX-7, Cat. AB0000456, UBC Ablab, 1:200. This monoclonal antibody has been validated by the original manufacturer by detection of PAX7 in chick embryos. For more information visit https://dshb.biology.uiowa.edu/PAX7.

Anti-Neurofilament heavy chain (Cat. #ab4680, Abcam). This polyclonal antibody has been validated by the manufacturer via detection of neurofilament heavy polypeptide in rat and mice brain sections. https://www.abcam.com/neurofilament-heavy-polypeptide-antibody-ab4680.html

Anti-Mouse SOX9 (Cat. #ab185966, Abcam). This recombinant monoclonal antibody recognizes mouse SOX9. https://www.abcam.com/sox9-antibody-epr14335-78-ab185966.html

Anti-Mouse CD31 (Cat. #550274, BD Pharmingen). This antibody recognizes mouse CD31 (PECAM1). https://www.bdbiosciences.com/ds/pm/tds/550274.pdf

Anti-Mouse MyHC (Cat. # M4276, Sigma Aldrich). This monoclonal antibody recognizes mouse Skeletal Myosin. https://www.sigmaaldrich.com/catalog/product/sigma/m4276?lang=en&region=CA&gclid=CjwKCAjwnef6BRAgEiwAgv8mQUX5WLA-rJSOw-LZMjhFHtrgHOSlTO2_x_QN5K58oJBvFA8GBuFc8hoC14kQAvD_BwE

Anti-RFP (Cat. # ab62341, Abcam). This polyclonal antibody reacts with tdTomato. https://www.abcam.com/rfp-antibody-ab62341.html

Anti-GFP (Cat. #ab13970, Abcam). This polyclonal antibody recognizes GFP. https://www.abcam.com/gfp-antibody-ab13970.html

Anti-COL22A1 (Cat. #ab121846, Abcam). This polyclonal antibody reacts with mouse COL22A1. https://www.abcam.com/col22a1-antibody-ab121846.html

Animals and other organisms

Policy information about studies involving animals; ARRIVE guidelines recommended for reporting animal research

Laboratory animals

Details on the mice used in this study have been included in the Methods section of this manuscript. All Hic1CT2; R26tdTom, B6.129S4-Pdgfratm11(EGFP)Sor/J , B6.Cg-Gt(ROSA)26Sortm14(CAG-tdTomato)Hze/J , and Ptch1tm1Mps/J mice were maintained in a C57Bl/6 background. Hic1nLacZ and Hic1CT2; R26tdTom; ScxGFP mice were maintained in a CD-1 background. For embryological experiments, both male and female embryos were used at specific ages (E10.5-18.5). The Ptch1tm1Mps/J mice were adult (8wk) males. The Hic1CT2; R26tdTom mice used for NTX-induced muscle injury were adult (8-10wk) males.

Wild animals

No wild animals were used in this study.

Field-collected samples

No field collected samples were used in this study.

Ethics oversight	Animals were maintained and experimental protocols were conducted in accordance with approved and ethical treatment standards of the Animal Care Committee at the University of British Columbia.

Note that full information on the approval of the study protocol must also be provided in the manuscript.

Flow Cytometry

Plots

Confirm that:

- [x] The axis labels state the marker and fluorochrome used (e.g. CD4-FITC).
- [x] The axis scales are clearly visible. Include numbers along axes only for bottom left plot of group (a 'group' is an analysis of identical markers).
- [x] All plots are contour plots with outliers or pseudocolor plots.
- [x] A numerical value for number of cells or percentage (with statistics) is provided.

Methodology

Sample preparation	Freshly dissected E11.5 and E12.5 Hic1CrERT2; R26tdTomato or Hic1CreERT2; R26tdTomato; ScxGFP embryo forelimbs were digested in a Dispase enzyme-based solution [Dispase II 10 units/ml; 10% fetal bovine serum in Puck's saline A]. Two-three forelimb pairs were pooled into 3 mL reactions. For E13.5, E14.5 and E16.5 forelimbs, the dissociation cocktail consisted of Collagenase A, Pronase, DNase in Dulbecco's Modified Eagle Medium (DMEM). Single forelimb pairs were dissociated in 2 ml reactions. In both cases, enzymatic digestion was carried out at 37C with gentle rotation for 1.5 hours with vortexing every 30 minutes. Digested material was then triturated by pipetting and passed through a 40 um cell strainer. The resultant cell suspension was washed with FACS buffer (2mM EDTA, 2% FBS in PBS) and centrifuged at 500 x g for 5 minutes. To further enrich for MPs, an antibody panel in combination with tdTomato fluorescence, was designed to label and exclude lineage-positive cells [anti-CD45-647, anti-CD117-APC, anti-CD31-APC, anti-CD11b-647, anti-F4/80-647, and anti-Ter119-647]. Cells were incubated in staining cocktail for 30 minutes on ice in the dark. Subsequently, cells were washed in 15 mls FACS buffer and centrifuged at 500 x g for 5 minutes. The cell pellet was resuspended in FACS buffer containing Hoechst 33342 and propidium iodide.
Instrument	A BD Influx instrument was used for all FACS enrichment. For flow cytometry, a BD LSR II was used.
Software	FACS data was acquired using BD FACS Sortware 1.0.0.650 and flow cytometry data was collected using BD FACS Diva 6.1.3. All analyses and visualizations were performed using FlowJo.
Cell population abundance	Post-sort FACS reanalysis was performed by the FACS technician to ensure successful enrichment of the relevant population. Identity of post-sort fractions was confirmed and the abundance of target cells was determined by fluorescence microscopy. Enriched target populations destined for single cell capture were further verified and enumerated by hemocytometer following the second sort as described in the Methods section of the text. The abundance of the target cell populations was almost 100% of post-sorted fractions.
Gating strategy	Forward and side scatter gating was determined empirically. All antibody lots were titrated for optimal separation. Negative and FMO controls were included in all experiments until the staining panel became routine. Hic1-lineage MPs were isolated as cells that were positive for tdTomato and negative for endothelial and hematopoietic lineage markers (CD31, Ter119, CD45, CD11b, F4/80, and CD117). For the EdU-incorporation assays, the tdTomato+ Lin- cells were further gated into tdTomato+ Lin- PacificBlue+ and tdTomato+ Lin- PacificBlue- cells by placing a quad gate in between the population density centers. This was facilitated by the use of contour plots as shown in Extended Data Figure 1e. For analysis of Hic1CT2; R26tdTom; ScxGFP forelimbs, mononuclear Lin- cells were classified into Lin- GFP+ and Lin- GFP- cells by gating at the lowest point of the histogram separating the two populations. The Lin- GFP+ cells were then further refined into tdTomato+ and tdTomato- fractions by placing a quad gate at the boundary between the two population density centers. Please see the accompanying Supplementary Information for a figure exemplifying the gating strategies described above.

- [x] Tick this box to confirm that a figure exemplifying the gating strategy is provided in the Supplementary Information.
